# Tree Plantation-Driven Forest Fragmentation Reduces Ground-Dwelling Insect Diversity Through Cascading Declines in Seedling Density

**DOI:** 10.3390/insects17040399

**Published:** 2026-04-07

**Authors:** Zhenyan Zhang, Chaoyou Jiang, Xinyu Zhu, Fengqun Meng

**Affiliations:** 1 Guangxi Key Laboratory of Forest Ecology and Conservation, School of Forestry, Guangxi University, Nanning 530004, China; 2 Guangxi State-Owned Bobai Forest Farm, Yulin 537600, China; 3 Experimental Centre of Tropical Forestry, Chinese Academy of Forestry, Pingxiang 532600, China; 4 Guangxi Colleges and Universities Key Laboratory for Cultivation and Utilization of Subtropical Forest Plantation, School of Forestry, Guangxi University, Nanning 530004, China

**Keywords:** biodiversity conservation, forest fragmentation, tree plantation, ground-dwelling insect

## Abstract

The global expansion of tree plantations has led to extensive fragmentation of natural forests, posing significant challenges for biodiversity conservation. Understanding the diversity patterns and underlying mechanisms of ground-dwelling insects in these fragmented landscapes is critical to inform effective conservation strategies. To address this, we sampled ground-dwelling insects using pitfall traps across nine remnant natural forest fragments (“islands”) embedded within a tree plantation matrix in Guangxi, China. We examined insect family-level diversity and community composition in relation to fragment isolation (low vs. high) and size (large vs. small) and explored the mechanisms driving the observed patterns. Our results revealed no significant difference in ground-dwelling insect diversity between low-isolation and high-isolation fragments. However, diversity was significantly lower in smaller fragments compared to larger ones. This reduction was primarily driven by decreased seedling density within smaller fragments, directly reflecting the adverse effects of plantation-driven fragmentation on native seedling establishment. Furthermore, we observed noble shifts in community composition of ground-dwelling insects along both fragment isolation and size gradients. Highly isolated fragments exhibited a decline in phytophagous insects and omnivores (with detritivore-herbivore diets), but an increase in detritivores. Smaller fragments exhibited consistent declines across multiple insect taxa spanning various dietary guilds. The observed changes in ground-dwelling insect composition were driven by shifts in plant (especially seedling) community composition. Our findings reveal a clear cascading effect: plantation-driven fragmentation limits native plant regeneration, and these limitations subsequently propagate to higher trophic levels, profoundly impacting ground-dwelling insects. Effective restoration of plantation-fragmented landscapes requires strategies that both prioritize the preservation of large, continuous forest fragments and promote native seedling recruitment within existing fragments.

## Simple Summary

Tree plantations are breaking up natural forests into smaller, isolated fragments, which harms biodiversity. We studied ground-dwelling insects in nine forest fragments surrounded by plantations in Guangxi, China. We found that smaller forest fragments had fewer types of insects compared to larger ones. This is because smaller fragments support fewer seedlings, making it harder for insect populations to thrive. We also observed changes in the types of insects present: in isolated fragments, some plant-feeding insects decreased, while decomposers—those that break down dead material—increased. In smaller fragments, multiple insect groups, including plant-eaters, omnivores, decomposers, and predators, declined. These changes reveal a ripple effect: when fragmentation harms plant regeneration, it does not stop there—this stress moves up the food chain, affecting insects and other wildlife to survive. To restore these fragmented landscapes, we need to both protect large, connected forest fragments and help native plants regenerate within existing fragments.

## 1. Introduction

Forest fragmentation—the process by which continuous forests are gradually divided into smaller, more isolated fragments—poses a great threat to global biodiversity. Today, most of the world’s remaining forests exist as small, highly isolated fragments [1,2]. Concurrently, tree plantations—primarily intensively managed monocultures of fast-growing tree species established for timber production (e.g., sawlogs, pulp) [3] and ecosystem services (e.g., carbon storage, soil erosion control, water provisioning) [4,5,6]—have expanded rapidly in recent decades. According to the Food and Agriculture Organization [7], the global area of tree plantations increased by more than 70%, from approximately 170 million hectares in 1990 to nearly 293 million hectares in 2020, with most growth occurring in biodiverse tropical and subtropical regions. However, this expansion has often come at the expense of natural forests, either through the direct conversion of native forests into tree plantations or by occupying land that could otherwise support natural forest regeneration. For example, the expansion of tree plantations is responsible for an estimated 7% of global tropical deforestation [8] and more than 50% of deforestation in parts of Southeast Asia [9]. Consequently, tree plantation expansion further fragments the already fragmented natural forests into even smaller, more isolated remnants. This pattern has been documented in regions including China [10,11], Malaysia [12], and Brazil [13], and is likely to intensify as plantations continue to expand. Despite this escalating threat, the biodiversity impacts of forest fragmentation driven by tree plantations remain poorly understood, especially when compared to fragmentation caused by non-forest land uses (e.g., agriculture, pasture, urbanization). This knowledge gap likely stems from the superficial resemblance of tree plantations to natural forests, which has led them to be overlooked as significant drivers of fragmentation. A thorough understanding of the impacts of tree plantation-driven forest fragmentation on biodiversity and their underlying mechanisms is vital for developing effective conservation strategies and policies to mitigate biodiversity loss and stabilize ecosystem services.

Insects, with millions of described and undescribed species, are the most species-rich taxonomic group on Earth. They provide crucial ecosystem services, including nutrient cycling, pollination, pest control, and supporting food webs [14]. However, widespread insect declines have become a critical global issue, with habitat fragmentation identified as a major driver [15,16]. However, our understanding of these declines is taxonomically and functionally biased. Research has overwhelmingly focused on a limited subset of conspicuous, morphologically identifiable groups, particularly such as Coleoptera (e.g., beetles) and Lepidoptera (e.g., butterflies and moths), as well as ecologically prominent functional guilds such as pollinators and herbivores [15,16,17,18,19,20,21]. While these studied taxa provide valuable insights, their dominance in the literature likely reflects methodological convenience rather than ecological representativeness, especially considering that many are volant and therefore less constrained by fine-scale landscape barriers. Conversely, ground-dwelling insects, which occupy the soil surface and the immediate aboveground layer (typically 0–15 cm), represent a crucial yet significantly understudied component of terrestrial insect diversity. They serve as essential biotic bridges between aboveground and belowground ecosystems, facilitating nutrient cycling, soil structuring, pest regulation, and seed dynamics [22]. Critically, their limited dispersal capacity makes them especially sensitive to environmental change [23,24], likely rendering them particularly vulnerable to the accumulation of physicochemical, biotic, and dispersal barriers resulting from habitat fragmentation.

Given the ongoing, yet underappreciated, forest fragmentation driven by tree plantations, and the hyperdiverse, functionally important yet sensitive nature of ground-dwelling insects, it is emergent and crucial to thoroughly understand the impacts of this fragmentation on their diversity and underlying mechanisms. Such knowledge is essential for informing effective conservation strategies and policies within these increasingly fragmented landscapes. However, to the best of our knowledge, only three studies have explicitly examined the impacts of tree plantation-driven forest fragmentation on ground-dwelling insects [25,26,27] and their findings are inconsistent. For example, coffee plantation-driven forest fragmentation reduced dung beetle diversity in Neotropical rainforests in Mexico [25]. In contrast, no effects of pine plantation-driven forest fragmentation were detected on carabid beetles in southeastern Australia [26], or on ground-dwelling beetles in temperate forests of northern China [27]. These discrepancies suggest that the impacts of plantation-driven fragmentation are highly context-dependent, varying across ecosystems and biomes. Consequently, current evidence remains limited and geographically restricted, preventing general conclusions about its broader effects on ground-dwelling insect diversity.

The theory of island biogeography (IBT) is frequently applied to evaluate species loss resulting from habitat fragmentation. In this context, remnant natural forest fragments are conceptualized as “islands” embedded within a “sea” of human-modified habitats (here, tree plantations) [28,29]. IBT posits that species diversity within these fragments is predominantly governed by their area and isolation, predicting that larger and less isolated fragments will harbor greater species diversity. Reduced species diversity in more isolated fragments is solely attributed to a decline in colonization rates due to increased isolation [30]. Several non-mutually exclusive mechanisms are proposed to explain why larger fragments tend to support higher species diversity. The area per se hypothesis (also known as ecological drift hypothesis), central to IBT, proposes that larger fragments can sustain larger population sizes, which inherently reduces local extinction rates, and often facilitates higher immigration, thereby maintaining higher species diversity at equilibrium [30]. The habitat quality hypothesis highlights the role of edge effects: smaller fragments exhibit a higher edge-to-interior ratio, rendering them more susceptible to intensified abiotic stresses (e.g., increased wind turbulence, desiccation, and microclimatic extremes) [31,32,33]. These stresses can disproportionately exclude habitat-sensitive specialist species. The habitat heterogeneity hypothesis argues that larger fragments encompass greater environmental variation and a wider array of microhabitats, consequently offering more ecological niches and thus supporting a broader range of species [34,35]. Finally, the passive sampling hypothesis posits that larger fragments may simply intercept more species by chance, reflecting a sampling artifact rather than ecological processes [34]. The hierarchical additive diversity partitioning framework is widely employed to disentangle these underlying mechanisms [36,37]. Under this approach, total species richness within a fragment (γ-diversity) is additively partitioned into two components: average within-plot diversity (α-diversity) and species turnover among plots (β-diversity). Each mechanistic hypothesis generates distinct and testable predictions within this framework. Specifically, the area per se and habitat quality hypotheses are predicted to increase γ-diversity primarily through elevated α-diversity in larger fragments. In contrast, the habitat heterogeneity hypothesis is expected to increase γ-diversity predominantly via increased β-diversity. In contrast, the passive sampling hypothesis predicts no systematic differences in either α- or β-diversity between large and small fragments.

In this study, we aimed to investigate how ground-dwelling insects respond to tree plantation-driven forest fragmentation. We hypothesized that larger and less isolated forest fragments would support higher overall ground-dwelling insect diversity (γ-diversity) compared to smaller and more isolated fragments. This expectation aligns with prior evidence indicating that many forest-dependent species are unable to persist in plantation matrices [24,38,39]. Moreover, similar negative effects of tree plantation-driven forest fragmentation have been documented for woody plants and soil microbes [38,39]. We further propose that larger and less isolated fragments would support higher α-diversity and β-diversity, with these patterns being primarily mediated by enhanced habitat quality and greater habitat heterogeneity, respectively. Our proposition is grounded in the understanding that tree plantation-driven forest fragmentation leads to significant reductions in plant diversity [38,39], which in turn can unleash profound cascading ecological effects on insect communities. More specifically, we anticipate that reduced plant diversity within smaller fragments will diminish both food resources and structural complexities. These reductions are predicted to impact insect diversity in two ways: the resulting degraded habitat quality (i.e., fewer food resources) will lead to reduced α-diversity, while the subsequent decrease in habitat heterogeneity (i.e., diminished structural complexities) is projected to result in lower β-diversity.

To test these hypotheses, we sampled ground-dwelling insects using pitfall traps at Yachang Forest Farm, Guangxi, China. Our sampling sites included nine remnant natural forest fragments (0.98–7.69 ha, characterized by similar stand structure and topography) embedded within a tree-plantation matrix, as well as a nearby continuous nature reserve and extensive monoculture plantations of *Eucalyptus* and *Pinus massoniana*. We quantified the three components of diversity (γ-, α-, and β-diversity) for ground-dwelling insects and examined how they varied in relation to fragment area and isolation. Additionally, we evaluated habitat quality and heterogeneity using a suite of environmental variables (vegetation, soil, and litter properties) and assessed their influences on α- and β-diversity.

## 2. Methods and Materials

### 2.1. Study Area

**Figure 1:**
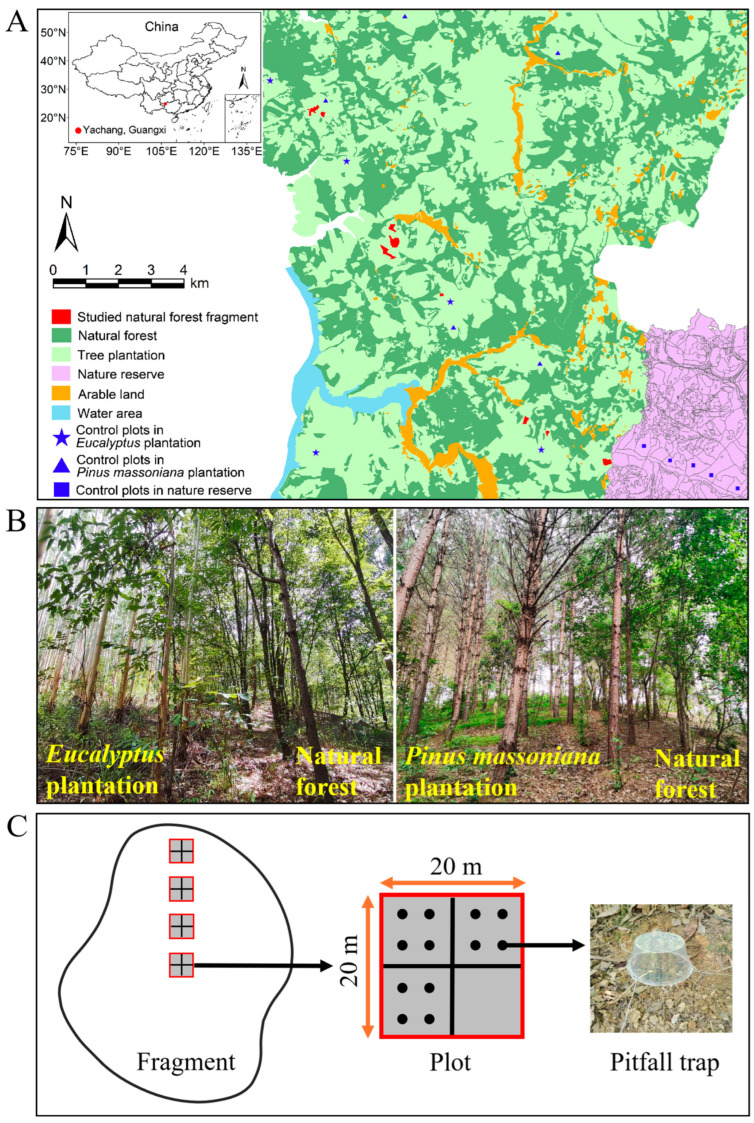
(**A**) Map illustrating natural forest fragmentation driven by tree plantations at Yachang Forest Farm, Guangxi, China; (**B**) photographs of the study sites highlighting the interface between tree plantations and natural forests; and (**C**) a schematic diagram of the sampling methodology employed in this study.

This study was conducted at Yachang Forest Farm (106°08′–106°25′ E, 24°36′–25°00′ N) in Leye County, Guangxi, China (Figure 1A). The study site is situated within the transitional zone between the southeastern margin of the Yunnan–Guizhou Plateau and Guangxi hilly basin, and is characterized by highly weathered Ultisols (i.e., “red soils”). The region experiences a subtropical monsoon climate, with a mean annual temperature of 16.3 °C, annual precipitation of 1058 mm (over 50% occurring in summer), and annual evaporation of 1485 mm. This climatic pattern often results in recurrent droughts during spring and autumn [40]. The landscape comprises a mosaic of secondary natural forests and managed tree plantations. These secondary forests are mid-successional communities that spontaneously developed from original primary *Pinus yunnanensis* var. *tenuifolia* stands following severe human-induced disturbances (e.g., wildfires, clear-cutting, and selective logging) during the 1960s–1970s. In these secondary stands, the canopy is typically dominated by oak species, mainly the evergreen *Cyclobalanopsis glauca* and the deciduous *Quercus variabilis*. The understory layer is characterized by shrub species such as *Cipadessa baccifera*, *Maesa japonica*, and *Callicarpa macrophylla*, while the forest floor is mainly covered by oak regeneration with sparse herbaceous vegetation [38].

### 2.2. Spatial Distribution of Remnant Forest Fragments

Remnant fragments were selected using data from the 2018 China Forest Management Planning Inventory dataset, provided by Guangxi Yachang Forest Farm. The study area features a heterogeneous mosaic landscape mainly dominated by two forest types: natural forests and tree plantations (Figure 1A). Natural forests exist in two configurations: (1) extensive, continuous intact natural forests, primarily within nature reserves, and (2) fragments embedded within a plantation matrix. Tree plantations, established since the 1990s on sites formerly occupied by natural forests, fall into three categories: (1) extensive, contiguous timber monocultures, which constitute 53.12% of the total tree plantation area, are predominantly composed of *Eucalyptus* spp. (33.47%), *P. massoniana* (13.39%), and *Cunninghamia lanceolata* (6.26%); (2) diverse economic plantations, accounting for 33.96% of the total tree plantation area, are sparsely scattered throughout the study region. These include tung oil, star anise, nut trees (e.g., chestnut, walnut), and fruit orchards (e.g., mango, plum, peach, citrus, longan); and (3) recently clear-cut areas, making up the remaining 12.92% of the total tree plantation area, are interspersed within the plantation landscape and are designated for future plantation establishment.

We initially identified 63 potential remnant forest fragments using ArcGIS software (version 10.8). A subsequent field survey led to the exclusion of 35 fragments due to inaccessible topography and another 11 due to significant human disturbance. We then selected the remaining 17 remnant forest fragments for ground-dwelling insect sampling. However, during the sampling period, eight of these fragments experienced severe damage to their pitfall traps by ants, necessitating their exclusion from further analysis. Ultimately, nine fragments remained for subsequent analyses, with sizes ranging from 0.98 to 7.69 ha (Figure 1A; Table S1). These fragments are characterized by oak-dominated stands, exhibiting an average canopy closure of 81%. They were situated in lowland areas (average slope: 26°; average elevation: 637 m) and embedded within a matrix of *Eucalyptus* and *P. massoniana* plantations (Figure 1B; Table S1). For analysis, these nine fragments were categorized by size into two groups: large fragments (>4 ha, *n* = 4) and small fragments (≤4 ha, *n* = 5) (Table S1). Fragment isolation was quantified by the distance to the nearby nature reserve (DNR). Similarly, fragments were grouped by isolation into low-isolation (DNR ≤ 8 km, *n* = 4) and high-isolation fragments (DNR > 8 km, *n* = 5) (Table S1). Given the lack of universally established ecological thresholds for fragment size and isolation pertinent to ground-dwelling insects, the 4-ha and 8-km thresholds were pragmatically chosen. This approach aimed to create two relatively balanced groups for both size and isolation, thereby facilitating robust comparative analysis and allowing for the exploration of potential ecological gradients.

### 2.3. Ground-Dwelling Insect Sampling

Insect inventories were conducted from July to August 2022. The experimental design adhered to the established methodology of our broader project investigating the impact of tree plantation-driven fragmentation on biodiversity [38,39]. Specifically, within each remnant forest fragment, three to five 20 × 20 m plots were established along an edge-to-core gradient, with the number of plots adjusted according to fragment size and topography (Figure 1C). In addition, control plots were established to enable a comprehensive assessment of insect diversity within the fragments [41]. Specifically, fifteen 20 × 20 m control plots were set up across the landscape (Figure 1A): five in large, continuous natural forests within the nature reserve (hereafter referred to as intact natural forests), five in *Eucalyptus* plantations, and five in *P. massoniana* plantations. Each plot was subdivided into four 10 × 10 m quadrats, three of which were randomly selected for ground-dwelling insect sampling. Within each selected quadrat, four pitfall traps were placed approximately 3 m apart (Figure 1C). Pitfall traps were constructed using disposable plastic cups, each 7.5 cm high and 7 cm in diameter. Into each trap, 100–150 mL of a mixed lure solution was added, composed of sugar, vinegar, alcohol, and water at a 1:2:1:20 volumetric proportion. To shield the traps from precipitation and potential inundation, a protective plastic bowl was suspended approximately 10 cm above each cup by means of an iron wire support. Traps were emptied every 10 days, with a total of five collections over the sampling period. Specimens from all traps within a plot, across the five collections, were pooled to form a single composite sample per plot, yielding a total of 51 samples for analysis. All specimens were preserved in 75% ethanol for subsequent identification.

Specimens were initially sorted into broad morphological categories in the laboratory using an online insect identification tool [42] and by comparing them with voucher material from the authors’ personal collections, which are deposited at Guangxi University. Afterward, specimens were sent to taxonomic specialists for further identification. Most specimens were identified by specialists from Beijing Dabu Biological Science and Technology Services Co., Ltd., Beijing, China, while Blattodea specimens were identified by experts from the Shanghai Entomological Museum, Shanghai, China. Identification was primarily based on morphological characteristics (e.g., wings, genitalia, body structure, legs, and chelicerae) and cross-referenced with standard taxonomic references, including Insect Taxonomy, Insect Classification, Chinese Insects Illustrated, Insects from Mt. Shiwandashan Area of Guangxi, and Blattodea of China [43,44,45,46,47,48]. Although a few common or dominant taxa were resolved to genus or species level, most identifications were made at the family level (see Table S2 for detailed information). Therefore, all insects were ultimately assigned to the family level for subsequent analyses to ensure consistency. We then determined the feeding habits for each family: for those identified to genus or species, the feeding habits of the dominant representative were used; for those identified solely to family, typical feeding habits for that family were ascertained from the online China Species Library [49].

### 2.4. Forest Characteristic Measurements

Within each selected quadrat, all woody plants were surveyed and categorized as trees (DBH ≥ 5 cm) or seedlings (DBH < 5 cm). Four evenly spaced sampling points were established within each selected quadrat (Figure 1C), where soil temperature, moisture content, and pH (measured in a 1:2.5 soil-deionized water suspension) were recorded using a portable environmental monitor (LD-QX07, LEADER, Shanghai, China). Litter cover and thickness were also visually estimated at each sampling point. Additionally, leaf litter was collected from a 50 × 50 cm area centered on each sampling point, and litter from the same quadrat was pooled to form a single composite sample. The litter samples were oven-dried at 65 °C for 48 h to constant mass and then ground to pass through a 1 mm sieve using a Wiley Mill. Litter carbon (C) content was determined by the combustion method using a C/N analyzer (Multi N/C 3100, Analytik Jena AG, Jena, Germany). Litter nitrogen (N) and phosphorus (P) contents were measured after H_2_SO_4_-H_2_O_2_ digestion, using a fully automatic flow-through analyzer (AA3, Bran Luebbe, Norderstedt, Germany) and the molybdenum-blue colorimetric method on a microplate reader (Infinite M200 PRO, TECAN, Männedorf, Switzerland), respectively. Litter potassium (K), calcium (Ca), and magnesium (Mg) contents were measured by atomic absorption spectrophotometry after H_2_SO_4_-H_2_O_2_ digestion. All environmental and nutrient variables were standardized (*z*-scored) to a mean of 0 and variance of 1 prior to statistical analyses.

### 2.5. Statistical Analysis

For each fragment, we assessed three diversity components (γ-, α-, and β-diversity) at the family level. γ-diversity, representing the total diversity within a fragment, was calculated as the total number of families present across all plots in each fragment. α-diversity was calculated as the mean family richness across all plots within a fragment. β-diversity was quantified as the mean pairwise Bray–Curtis dissimilarity among plots within a fragment using the “vegdist” function in the R package vegan [50]. To control sampling effects, γ-, α-, and β-diversity metrics were also computed after standardizing the number of plots to three per fragment. Differences in γ-, α-, and β-diversity between large and small fragments, as well as between low-isolation and high-isolation fragments, were assessed using *t*-tests. Given the significant differences in α-diversity between large and small fragments, stepwise multiple linear regression (SMLR) was then performed to identify the optimal combination of predictors for α-diversity. Candidate independent variables included woody plant community characteristics (density and diversity) as well as soil and litter properties. Model selection was conducted by sequentially removing each predictor and evaluating the resulting changes in Akaike’s information criterion (AIC) and the coefficient of determination (*R*^2^). Predictors were excluded from the model if their removal caused negligible changes in AIC (ΔAIC < 2) or *R*^2^ (Δ*R*^2^ < 0.01) [51,52]. The final model was then refitted using ordinary multiple linear regression, and the relative importance of the retained predictors was quantified using the “lmg” function in the R package relaimpo [53].

We performed permutational multivariate analysis of variance (PERMANOVA) to evaluate whether ground-dwelling insect community composition at the family level differed significantly between large and small fragments, as well as between low-isolation and high-isolation fragments. Furthermore, when significant differences in community composition were detected, similarity percentage analysis (SIMPER) was conducted to identify the families that significantly contributed to the dissimilarity between these fragment categories. PERMANOVA and SIMPER analyses were performed using the “adonis” and “simper” functions, respectively, in the vegan package [50]. To visualize compositional differences, non-metric multidimensional scaling (NMDS) was conducted using the “metaMDS” function in the vegan package. Distance-based SMLR was performed to identify the optimal set of predictors for ground-dwelling insect community composition, following the same selection criteria as above. Independent variables included woody plant community composition, soil properties, and litter properties. We quantified dissimilarities using pairwise Euclidean distances for soil and litter properties, and Bray–Curtis distances for insect and plant community compositions. All distance matrices were generated using the “vegdist” function in the vegan package [50].

## 3. Results

### 3.1. Characteristics of Ground-Dwelling Insect Communities

A total of 15,495 individuals, representing 8 orders and 67 families, were collected across all plots using pitfall traps (for detailed counts of each order, see Table S3). At the order level, ground-dwelling insect communities across all plots were consistently dominated by Blattodea (mean ± SE: 42.1 ± 2.5%), Orthoptera (19.1 ± 2.4%), Diptera (15.9 ± 2.2%), and Coleoptera (13.3 ± 1.3%), followed by Hemiptera (8.6 ± 1.2%), Dermaptera (0.4 ± 0.1%), Hymenoptera (0.4 ± 0.1%), and Lepidoptera (0.2 ± 0.1%), although relative abundances varied among plots (Figure S1).

### 3.2. Effects of Fragment Isolation and Area on Forest Characteristics

Forest characteristics responded differentially to fragment isolation (Figure S2). In terms of soil properties, soil pH was comparable between low-isolation and high-isolation fragments (Figure S2A). However, low-isolation fragments exhibited significantly higher soil moisture content and significantly lower soil temperature (Figure S2B,C). For litter nutrient contents, no significant differences were observed in C, P, K, Ca, or Mg contents between low-isolation and high-isolation fragments (Figure S2D–H). In contrast, N content was significantly higher in low-isolation fragments than in high-isolation fragments (Figure S2I). Litter cover and thickness did not differ significantly between low-isolation and high-isolation fragments (Figure S2J,K). With respect to forest stand structure, tree and seedling densities showed no significant differences between low-isolation and high-isolation fragments (Figure S2L,M). However, both tree and seedling species richness were significantly higher in low-isolation fragments compared to high-isolation fragments (Figure S2N,O). Tree (PERMANOVA: *F* = 3.89, *R*^2^ = 0.089, *p* = 0.001; Figure S3A) and seedling (PERMANOVA: *F* = 6.58, *R*^2^ = 0.141, *p* = 0.001; Figure S3B) community compositions differed significantly between low-isolation and high-isolation fragments.

Similarly, forest characteristics exhibited differential responses to fragment area (Figure S4). Regarding soil properties, soil pH did not differ significantly between large and small fragments (Figure S4A). However, soil moisture content was significantly higher, and soil temperature was significantly lower in large fragments than in small fragments (Figure S4B,C). For litter nutrient contents, no significant differences were detected in C, P, Ca, or Mg contents between large and small fragments (Figure S4D–G). In contrast, N content was significantly lower, whereas K content was significantly higher, in large fragments compared to small fragments (Figure S4H,I). Additionally, litter cover was significantly greater in small fragments than in large fragments, while litter thickness did not differ significantly between large and small fragments (Figure S4J,K). Regarding forest stand structure, seedling density was significantly higher in large fragments than in small fragments, whereas tree density showed no significant difference (Figure S4L,M). Furthermore, neither tree nor seedling species richness differed significantly between large and small fragments (Figure S4N,O). However, tree (PERMANOVA: *F* = 3.66, *R*^2^ = 0.084, *p* = 0.001; Figure S5A) and seedling (PERMANOVA: *F* = 2.58, *R*^2^ = 0.061, *p* = 0.002; Figure S5B) community compositions differed significantly between large and small fragments.

### 3.3. Differences in Diversity and Community Composition of Ground-Dwelling Insects Between Forest Fragments and Tree Plantations, and Between Forest Fragments and Intact Natural Forest

We conducted *t*-tests to assess differences in γ-, α-, and β-diversity between forest fragments and the three reference forest types (i.e., intact natural forests, *Eucalyptus* plantations, and *P. massoniana* plantations). For γ-diversity, large fragments (with one exception) exhibited significantly higher γ-diversity than both intact natural forests and *Eucalyptus* plantations, whereas small fragments showed significantly lower γ-diversity than these two forest types (Figure S6A). All fragments except one showed higher γ-diversity than *P. massoniana* plantations (Figure S6A). For α-diversity, large fragments exhibited higher or comparable values relative to the three reference forest types, whereas small fragments showed lower or comparable α-diversity (Figure S7A). For β-diversity, no significant differences were detected between intact natural forests and fragments, or between *Eucalyptus* plantations and fragments, except for one fragment with lower β-diversity (Figure S8A). Compared with *P. massoniana* plantations, five of the nine fragments showed similar β-diversity, while the remaining four exhibited significantly higher β-diversity (Figure S8A). These patterns remained consistent after controlling for sampling effects by standardizing the number of plots to three per fragment or forest type (Figures S6B, S7B, and S8B). The community composition of ground-dwelling insects varied significantly among forest fragments, intact natural forests, *Eucalyptus* plantations, and *P. massoniana* plantations, with significant differences observed between any pair of forest types (PERMANOVA: *F* = 8.19, *R*^2^ = 0.316, *p* = 0.001; Figure S9). Forest fragments were dominated (relative abundance ≥ 10%) by Blattellidae (37.0 ± 2.8%), Gryllidae (19.3 ± 3.0%), Drosophilidae (13.2 ± 2.4%) and Scarabaeidae (9.4 ± 1.7%) (Figure S10). Intact natural forests were dominated by Drosophilidae (29.6 ± 7.7%), Reduviidae (16.3 ± 2.7%), Blattidae (13.9 ± 2.7%) and Gryllidae (12.2 ± 3.3%) (Figure S10). *P. massoniana* plantations were primarily dominated by Gryllidae (28.5 ± 2.8%), Blattidae (26.9 ± 2.9%), and Reduviidae (26.7 ± 4.5%) (Figure S10). *Eucalyptus* plantations were primarily dominated by Blattidae (51.8 ± 3.7%) (Figure S10).

### 3.4. Effects of Fragment Isolation and Area on Ground-Dwelling Insect Diversity

**Figure 2:**
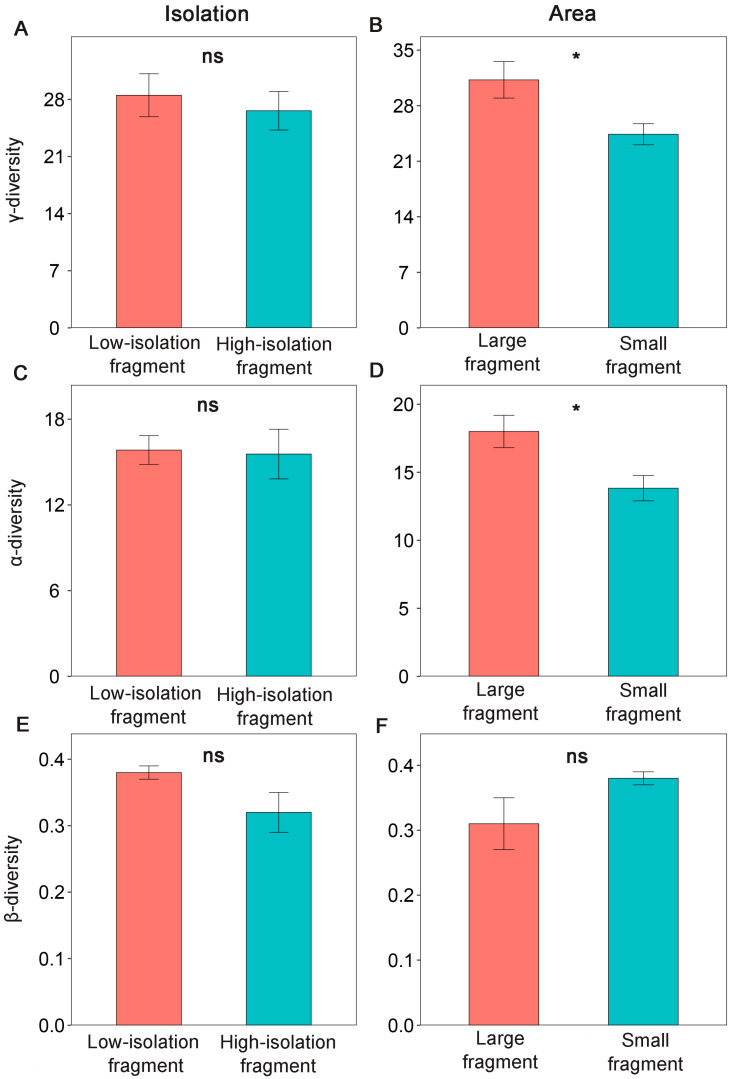
Effects of fragment isolation (**A**,**C**,**E**) and area (**B**,**D**,**F**) on γ-, a-, β-diversity of ground-dwelling insects in nine remnant natural forest fragments surrounded by tree plantations at Yachang Forest Farm, Guangxi, China. ns indicates no significant difference between fragment categories, *: *p* < 0.05. *n* = 4 and 5 for low-isolation and high-isolation fragments, respectively. *n* = 4 and 5 for large and small fragments, respectively.

No significant differences in γ-, α-, or β-diversity were observed between low-isolation and high-isolation fragments (Figure 2A,C,E). These patterns remained consistent after controlling for sampling effects by standardizing the number of plots to three per fragment (Figure S11A,C,E).

In contrast, γ-diversity was significantly higher in large fragments compared to small ones (Figure 2B). Similarly, α-diversity was also significantly greater in large fragments (Figure 2D), whereas β-diversity did not differ significantly between large and small fragments (Figure 2F). These trends remained consistent after controlling for sampling effects by standardizing the number of plots to three per fragment (Figure S11B,D,F).

SMLR was then performed to identify the optimal set of predictors of α-diversity. Analyses were conducted separately for each plant metric (density and species richness). When plant density was used as the plant metric, removal of soil pH, soil moisture content, soil temperature, litter C, litter N, litter P, litter K, litter Ca, litter cover, or litter thickness had negligible effects on AIC (ΔAIC < 2). In contrast, removing litter Mg, tree density, or seedling density led to a marked increase in AIC and a reduction in model *R*^2^ (Table 1). However, subsequent multiple linear regression analysis revealed that neither litter Mg (*p* = 0.505) nor tree density (*p* = 0.097) contributed significantly to the model. Consequently, seedling density emerged as the primary driver of α-diversity, explaining 26.2% of the variation and exhibiting a significant positive relationship (Table 2). When species richness was used as the plant metric, none of the measured variables made a significant contribution to the model (Tables S4 and S5).

### 3.5. Effects of Fragment Isolation and Area on Ground-Dwelling Insect Community Composition

**Figure 3:**
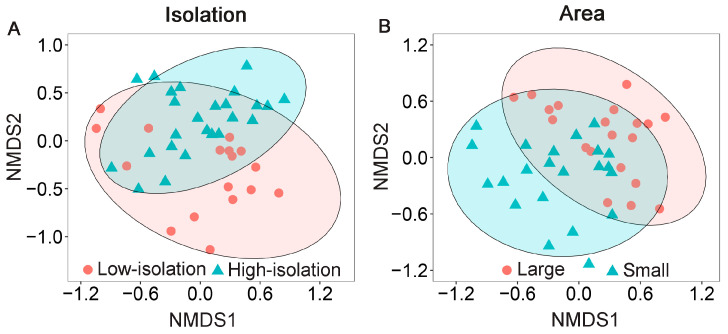
Non-metric multidimensional scaling (NMDS) ordination illustrating the effects of fragment isolation ((**A**): low vs. high isolation) and fragment area ((**B**): large vs. small) on the community composition of ground-dwelling insects in nine remnant natural forest fragments embedded within tree plantations at Yachang Forest Farm, Guangxi, China. Red circles denote 95% confidence ellipses for low-isolation fragments (A) and large fragments (B). Blue circles denote 95% confidence ellipses for high-isolation fragments (A) and small fragments (B).

With respect to fragment isolation, the community composition of ground-dwelling insects differed significantly between low-isolation and high-isolation fragments (PERMANOVA: *F* = 6.82, *R*^2^ = 0.146, *p* = 0.001; Figure 3A). SIMPER analysis identified 11 taxa that significantly contributed to this compositional dissimilarity. In contrast, 56 insect taxa showed no significant differences between low-isolation and high-isolation fragments, with Blattellidae, Scarabaeidae, and Reduviidae present in all plots. Of the 11 significant contributors, seven taxa were designated as key contributors (each contributing ≥ 1% to the dissimilarity), collectively explaining 68.3% of the compositional dissimilarity (Table 3). Among these key contributors, Blattellidae (more abundant in high-isolation fragments) and Drosophilidae (more abundant in low-isolation fragments) were the primary contributors, accounting for 33.1% and 18.0% of the compositional dissimilarity, respectively. Scarabaeidae (more abundant in high-isolation fragments) followed, contributing 8.0%. The remaining four taxa each contributed less than 3.3% to the compositional dissimilarity: Blattidae was more abundant in high-isolation fragments, whereas Nitidulidae, Staphylinidae, and Reduviidae were more abundant in low-isolation fragments. More detailed information on these key contributors is provided in Table 3. The other four significant contributors (each with a relative abundance of <0.01) were classified as minor contributors, each explaining less than 1% and together accounting for 1.2% of the compositional dissimilarity. Among these minor contributors, Anisolabididae was significantly more abundant in low-isolation fragments compared to high-isolation fragments, while Laemophloeidae and Cicadidae were completely absent from high-isolation fragments. In contrast, Polyphagidae was significantly less abundant in low-isolation fragments.

With respect to fragment area, the community composition of ground-dwelling insects also differed significantly between large and small fragments (PERMANOVA: *F* = 5.87, *R*^2^ = 0.128, *p* = 0.001; Figure 3B). SIMPER analysis identified 30 taxa that significantly contributed to this compositional dissimilarity. In contrast, 37 insect taxa showed no significant differences between small and large fragments, with Blattellidae, Scarabaeidae, and Reduviidae present in all plots. Of the 30 significant contributors, six taxa were identified as key contributors, each explaining more than 1% and collectively explaining 76.5% of the compositional dissimilarity (Table 4). Among these key taxa, Blattellidae, Gryllidae, and Drosophilidae were the primary contributors (each contributing >16% to the dissimilarity), while Phoridae, Mordellidae, and Staphylinidae made minor contributions (each accounting for <2% of the dissimilarity). All six key taxa were more abundant in large fragments than in small fragments. More detailed information on these key contributors is provided in Table 4. The remaining 24 significant contributors (each with a relative abundance of <0.01) were classified as minor contributors, each explaining less than 1% and collectively accounting for 2.9% of the compositional dissimilarity. Consistent with the key contributors, all these minor contributors were significantly more abundant in large fragments. This included taxa such as Coreidae, Catantopidae, Polyphagidae, Elateridae, Acrididae, Lauxaniidae, Pentatomidae, and Laemophloeidae. Furthermore, 16 of these minor taxa, including Dolichopodidae, Ichneumonidae, Sciaridae, Lygaeidae, Myrmecophilidae, Cercopidae, Lucanidae, Eulophidae, Tetrigidae, Apidae, Cerambycidae, Elasmidae, Anthomyiidae, Empididae, Milichiidae, and Muscidae, were entirely absent from small fragments.

Distance-based SMLR was conducted to identify the optimal set of predictors for ground-dwelling insect community composition. Removal of soil pH, soil moisture content, or litter C resulted in a significant increase in AIC (ΔAIC > 2) but caused only a minimal loss of explanatory power (Δ*R*^2^ < 0.01; Table 1). Removal of soil temperature, litter P, litter Ca, litter Mg, litter cover, or litter thickness had a negligible effect on AIC or model *R*^2^ (Table 1). However, removing litter N, litter K, tree community composition, or seedling community composition notably increased AIC and decreased model *R*^2^ (Table 1). Consequently, litter N, litter K, tree community composition, and seedling community composition were identified as the key drivers of ground-dwelling insect community assembly. Subsequent multiple linear regression analysis revealed that these four predictors collectively explained 24.2% of the variation in ground-dwelling insect community composition (Table 2). Among these, seedling community composition and tree community composition were the dominant predictors, accounting for 12.5% and 8.6% of the variation, respectively, while litter N and litter K exerted weaker but still significant effects, explaining 1.6% and 1.5% of the variation, respectively.

## 4. Discussion

Contrary to our hypothesis, we observed no statistically significant difference in γ-diversity (i.e., fragment-scale diversity) of ground-dwelling insects between low-isolation and high-isolation fragments. However, as predicted, γ-diversity was significantly lower in small fragments than in large ones. Nevertheless, we detected notable shifts in community composition of ground-dwelling insects along both fragment size and isolation gradients. Taken together, these results suggest that tree plantation-driven forest fragmentation exerts substantial negative effects on ground-dwelling insect assemblages. Our findings align with meta-analytic and synthetic evidence that highlights the pervasive negative impacts of habitat fragmentation on insect (mainly pollinators and herbivores) diversity [15,16]. However, due to the limited number of studies on the effects of habitat fragmentation on ground-dwelling insects, a clear consensus on the direction and magnitude of these effects remains elusive. Previous research (primarily on dung beetles and carabids) suggests that forest fragmentation effects on ground-dwelling insects are strongly contingent on the characteristics of the surrounding matrix [54]. Smoother contrasts between fragments and the matrix typically result in no detectable effects on ground-dwelling beetles [17,18,19,20]. In contrast, sharp contrasts impede dispersal and thereby amplify the negative consequences of fragmentation [20,21]. For example, small and highly isolated fragments embedded in highly intensive agricultural matrices exhibited reduced dung beetle species richness in the Brazilian Atlantic Forest [20]. Conversely, no significant fragment effects on ground-dwelling insect diversity were observed in the Chaco Serrano forests of Argentina, where fragments are surrounded by a relatively permeable, low-intensity agricultural matrix [17]. More importantly, even when the fragments and the surrounding matrix superficially resemble each other (e.g., both being tree-covered), differences in vegetation structural complexity (e.g., single-layered monocultures versus multi-layered, diverse forests) can still generate pronounced negative fragmentation effects. For example, negative impacts of forest fragmentation on dung beetle diversity were reported in multi-layered, diverse Neotropical rainforest fragments surrounded by monoculture coffee plantations in Mexico [25]. Conversely, no significant fragmentation effects were observed for carabid beetles in native single-layered *Eucalyptus* fragments embedded within monoculture pine plantations in southeastern Australia [26], nor for ground-dwelling beetles in native single-layered *Quercus liaotungensis* fragments embedded within monoculture pine plantations in temperate deciduous forests of northern China [27]. In the context of our study, monoculture tree plantations supported ground-dwelling insect communities that differed markedly from those in intact natural forests, suggesting that such plantations function more as ecological barriers than as dispersal corridors. Thus, although monoculture plantation matrices may superficially resemble natural forests, they can impose strong fragmentation effects on structurally complex native forests, ultimately leading to declines in ground-dwelling insect diversity. Taken together with previous studies, we underscore that significant fragmentation effects can arise not only from pronounced superficial contrasts between native fragments and the matrix, but also, and perhaps more insidiously, from differences in vegetation structural complexity, even when the matrix and fragments outwardly resemble each other.

### 4.1. Mechanisms Underlying the Effects of Fragment Isolation on Ground-Dwelling Insect Diversity

The absence of a significant difference in ground-dwelling insect diversity between low-isolation and high-isolation fragments, together with a pronounced shift in community composition in highly isolated fragments, likely reflects a balance between two opposing processes. Fragment isolation limits the colonization of forest-specialist ground-dwelling insects from the nearby natural reserve, whereas the permeability of the surrounding matrix facilitates the influx of insects from plantation habitats into the fragments. Specifically, on the one hand, plantations are not entirely inhospitable to ground-dwelling insects, as several taxa were present across all fragments and tree plantations. These include the highly omnivorous detritivore Blattellidae (dominated by *Blattella germanica*), the highly mobile detritivore Scarabaeidae (dominated by *Onthophagus vacca*), and the highly mobile generalist predator Reduviidae (dominated by *Rhynocoris annulatus*). Their presence indicates that these insects can disperse from nearby nature reserves, partially mitigating the effects of isolation. However, significant differences in the community composition of ground-dwelling insects have been observed between tree plantations and adjacent natural forests [24,55], as well as between high-isolation and low-isolation fragments. This suggests that fragments are at least somewhat isolated from the colonization source (i.e., the nearby nature reserve), leading to a decline in forest-specialist ground-dwelling insects. On the other hand, the surrounding plantation matrix serves as an alternative colonization source. Some insects also invade from the surrounding plantation matrix, contributing to an overall increase in species richness and masking differences between isolation levels. Consistent with these expectations, certain phytophagous taxa, such as Laemophloeidae and Cicadidae (Table S2), were completely absent from high-isolation fragments, likely due to their reliance on specific host plants. In addition, highly isolated fragments also exhibited significant declines in omnivores with a detritivore-herbivore diet, such as Drosophilidae (dominated by *Drosophila immigrans*) and Nitidulidae (dominated by *Omosita depressa*), and their predators, such as Anisolabididae (dominated by *Euborellia annulipes*), Staphylinidae (dominated by *Paederus fuscipes*) and Reduviidae (dominated by *Rhynocoris annulatus*) (Table S2). These taxa are known to dominate natural forests yet are considerably less prevalent in plantations across Guangxi (including our study site), China [24]. Concurrently, the ecological niches vacated by these declines may facilitate colonization by insects from the surrounding plantation matrix, particularly detritivorous groups, such as Blattellidae (dominated by *Blattella germanica*), Blattidae (dominated by *Blatta orientalis*), Scarabaeidae (dominated by *Onthophagus vacca*), and Polyphagidae (dominated by *Eupolyphaga sinensis*) (Table S2), which are characteristic of both *P. massoniana* [24] and *Eucalyptus* plantations [55]. Crucially, we further found that the observed shift in ground-dwelling insect community composition in highly isolated fragments relative to low-isolation fragments was driven by concurrent changes in plant community composition. This suggests that the effects of fragment isolation on ground-dwelling insect communities are not solely driven by altered dispersal dynamics but are further amplified by cascading changes in vegetation.

### 4.2. Mechanisms Underlying the Effect of Fragment Area on Ground-Dwelling Insect Diversity

The observed reduction in γ-diversity in small fragments was primarily driven by lower α-diversity rather than β-diversity. At first glance, this pattern appears consistent with the predictions of the area per se effect, which posits that lower α-diversity in small fragments can result from a neutral equilibrium between species immigration and extinction, as suggested by IBT [30]. However, our results suggest that fragment area influenced α-diversity mainly through its effect on seedling density, thereby supporting the habitat quality hypothesis. Meanwhile, we detected a pronounced shift in ground-dwelling insect community composition in small fragments relative to large ones, characterized by consistent declines in the relative abundances of 30 insect taxa with diverse dietary preferences in smaller fragments. This shift was strongly associated with changes in the woody plant (particularly seedling) community composition. Taken together, our findings highlight that reductions in seedling density are the primary driver of declines in ground-dwelling insect diversity. Indeed, seedling density in small fragments was only 68% of that in large fragments. Furthermore, our previous studies in this system have shown that while long-lived adult trees in remnant fragments may persist for several generations after fragmentation due to extinction debt, significant loss of seedling species due to establishment limitations has already occurred, even within the relatively short (around 30 years) isolation history of this region [38]. Consequently, we deduce that tree plantation-driven forest fragmentation negatively impacted seedling establishment, which in turn triggered cascading effects on ground-dwelling insect communities. Reduced seedling establishment in small fragments can lower ground-dwelling insect diversity via several mechanisms. First, it leads to a direct loss of food resources. Declines in seedling density reduce the availability of food resources (e.g., leaves, flowers, and fruits), resulting in decreases or local extinctions of phytophagous insects. Consistent with this mechanism, we observed reduced relative abundances of phytophagous insect taxa, such as Coreidae (dominated by *Homoeocerus* sp.), Catantopidae (dominated by *Xenocatantops humilis*), Elateridae (dominated by *Ampedus sanguinolentus*), Acrididae (dominated by *Phlaeoba infumata*), Pentatomidae (dominated by *Rhacognathus punctatus*), and Laemophloeidae, and even the complete absence of certain phytophagous insect taxa that likely depend on specific host plants, such as Lygaeidae (dominated by *Metochus abbreviates*), Cercopidae, Lucanidae, Tetrigidae, Apidae, Cerambycidae, and Empididae in small fragments (Table S2). Second, reduced seedling density likely leads to a loss of microhabitats. Although seedling density significantly affected the α-diversity of ground-dwelling insects, the α-diversity was not significantly related to either tree richness or seedling richness. This pattern suggests that seedlings influence insect communities not only as food resources but also by enhancing structural complexity and microhabitat availability. Consequently, lower seedling density in small fragments likely reduces habitat heterogeneity, leading to declines in omnivorous taxa, such as Mordellidae (dominated by *Scirtes* sp.), Phoridae (dominated by *Megaselia scalaris*), Drosophilidae (dominated by *Drosophila immigrans*), Anthomyiidae, Muscidae, and Lauxaniidae (dominated by *Homoneura euaresta*), as well as detritivorous taxa, such as Blattellidae (dominated by *Blattella germanica*), Gryllidae (dominated by *Loxoblemmus arietulus*), Sciaridae, Milichiidae, and Polyphagidae (dominated by *Eupolyphaga sinensis*) (Table S2). Ultimately, reductions in phytophagous, omnivorous, and detritivorous insects are expected to cascade upward, leading to declines in their predators, such as Staphylinidae (dominated by *Paederus fuscipes*), and even the complete absence of certain predator groups, such as Myrmecophilidae, Dolichopodidae, Ichneumonidae (dominated by *Xanthopimpla* sp.), Eulophidae, and Elasmidae (Table S2). The predominant role of understory vegetation in shaping ground-dwelling insect assemblages has been documented in previous studies. For example, Albacete et al. [23] found that vegetation structure (particularly understory cover) was the key driver of ground arthropod communities in non-riparian chestnut forests. Similarly, Wei et al. [24] demonstrated that understory tree density was the primary factor influencing ground-dwelling insect assemblages in *P. massoniana* plantations.

## 5. Conclusions

A key caveat of this study is that its reliance on family-level identification and inferring feeding habits from dominant species or genera within each family likely underestimates true biodiversity loss and potentially obscure the precise mechanisms by which tree plantation-driven forest fragmentation affects ground-dwelling insects. While species-level taxonomic resolution undoubtedly offers greater precision and deeper ecological insight, its implementation is often hindered by inherent practical challenges of morphological identification. These include the vast number of undescribed species, intricate morphology, small body sizes, limited diagnostic keys, and a general lack of taxonomic expertise [56,57]. Consequently, species-level identification is often only feasible for selected taxa or through advanced methods like DNA barcoding. However, phylogenetic conservatism often leads to functional redundancy within families. This allows family-level metrics to reliably detect ecological responses to disturbance, comparable to species-level data, across diverse arthropod groups (e.g., spiders, beetles, butterflies, moths, mites) [58,59]. In this context, family-level diversity effectively captures functional diversity and represents a practical trade-off. It offers a realistic, cost-effective, and methodologically robust alternative, particularly among poorly known groups, without sacrificing sensitivity to human-induced impacts [59]. Indeed, family-level metrics are a widely accepted and practical approach in studies of insect diversity within plantation systems [58,60,61,62] and in fragmented forest landscapes [19].

Although there was no significant difference in ground-dwelling insect diversity between low-isolation and high-isolation fragments, their community composition shifted markedly. Highly isolated fragments showed pronounced declines in phytophagous insects (with some taxa completely absent) and detritivore-herbivore omnivores, along with increases in detritivores. In addition, smaller fragments harbored lower diversity than larger ones, with consistent declines in the abundances of various insect taxa across diverse dietary groups, particularly phytophagous insects and predators (especially parasitoids), some of which were entirely absent. These results demonstrate that tree plantation-driven forest fragmentation exerts substantial negative effects on ground-dwelling insect communities. Importantly, this negative effect was mediated by reduced seedling availability—a consequence of fragmentation impeding seedling establishment—which indirectly triggered cascading effects and substantially reduced ground-dwelling insect diversity. Our findings have important implications for biodiversity conservation in fragmented forests. Although small fragments may retain conservation value [63], protecting and restoring large, continuous natural forest fragments should remain a priority, as they serve as irreplaceable reservoirs for sensitive forest specialist species. Restoration efforts within existing fragments should focus on re-establishing native understory vegetation and actively promoting native seedling recruitment. Given the taxonomic limitations of the present study, further species-level assessments, likely via DNA barcoding, are needed to better quantify insect diversity responses to tree plantation-driven fragmentation and inform effective conservation strategies.

Consistent with our findings, similar negative effects of tree plantation–driven forest fragmentation have also been reported for woody plants and soil microbes within the same study system [38,39], as well as for birds [64,65,66], small mammals [66,67], dung beetles [25], and butterflies [68] in other ecosystems. Taken together, these studies suggest that, although tree plantations may superficially resemble natural forests, plantation-driven forest fragmentation imposes substantial negative impacts on overall biodiversity. Reforestation with mixed-species plantations, rather than monocultures, therefore represents a crucial and emergent strategy for long-term biodiversity conservation.

## Figures and Tables

**Table 1:** Summary of stepwise regression models explaining variation in α-diversity and community composition of ground-dwelling insects in nine remnant natural forest fragments embedded within tree plantations at Yachang Forest Farm, Guangxi, China. Plant metrics were represented by tree and seedling densities.

Variable	α-Diversity			Community Composition		
	*R* ^2^	Δ*R*^2^	ΔAIC	*R* ^2^	Δ*R*^2^	ΔAIC
Full model	0.583	0	0	0.261	0	0
-Soil pH	0.583	0.000	−1.96	0.256	0.005	3.78
-Soil moisture	0.562	0.021	0.06	0.255	0.006	4.32
-Soil temperature	0.574	0.009	−1.10	0.260	0.001	−1.48
-Litter C	0.571	0.012	−0.82	0.254	0.007	5.30
-Litter N	0.546	0.037	1.59	0.239	0.022	22.78
-Litter P	0.561	0.022	0.19	0.260	0.001	−1.51
-Litter K	0.574	0.009	−1.08	0.246	0.015	14.35
-Litter Ca	0.583	0.000	−1.92	0.259	0.002	−0.56
-Litter Mg	0.538	0.045	2.31	0.260	0.001	−1.52
-Litter cover	0.55	0.033	1.22	0.261	0.000	−1.99
-Litter thickness	0.577	0.006	−1.34	0.257	0.004	1.62
-Tree density	0.449	0.134	9.75	/	/	/
-Seedling density	0.458	0.125	9.04	/	/	/
-Tree community	/	/	/	0.228	0.033	35.43
-Seedling community	/	/	/	0.182	0.079	85.45

Note: The full model included all explanatory variables. Symbols are defined as follows: “-” in the first column indicates a variable excluded by stepwise selection; “/” indicates a variable that is not applicable as a predictor; ΔAIC (Akaike information criterion) and Δ*R*^2^ represent the differences in AIC and model *R*^2^, respectively, relative to the full model.

**Table 2:** Summary of ordinary multiple linear regression models testing the effects of final selected explanatory variables on the α-diversity and community composition of ground-dwelling insects in nine remnant natural forest fragments embedded within tree plantations at Yachang Forest Farm, Guangxi, China.

Variable	Estimate	SE	*t*	*p*	Partial *R*^2^
α-diversity: *R*^2^ = 0.262, *p* < 0.001					
Seedling density	0.51	0.14	3.77	<0.001	0.262
Ground-dwelling insect community: *R*^2^ = 0.242, *p* < 0.001					
Litter N	−0.03	0.01	−5.5	<0.001	0.016
Litter K	0.02	0.01	3.62	<0.001	0.015
Tree community	0.24	0.04	6.35	<0.001	0.086
Seedling community	0.42	0.04	9.57	<0.001	0.125

**Table 3:** Key contributors and their contributions to the dissimilarity in community composition of ground-dwelling insects between low-isolation (*n* = 4) and high-isolation (*n* = 5) fragments embedded within tree plantations at Yachang Forest Farm, Guangxi, China. Different letters within the same row indicate significant differences in relative abundance between low-isolation and high-isolation fragments (*p* < 0.05). Data are presented as mean ± SE.

Order	Family	Contribution (%)	Relative Abundance	
			Low-Isolation Fragment	High-Isolation Fragment
Blattodea	Blattellidae	33.1	0.251 ± 0.027 b	0.452 ± 0.035 a
	Blattidae	2.1	0.028 ± 0.007 b	0.032 ± 0.005 a
Diptera	Drosophilidae	18.0	0.256 ± 0.040 a	0.048 ± 0.015 b
Coleoptera	Scarabaeidae	8.0	0.050 ± 0.010 b	0.125 ± 0.027 a
	Nitidulidae	2.5	0.044 ± 0.008 a	0.010 ± 0.002 b
	Staphylinidae	1.3	0.022 ± 0.005 a	0.005 ± 0.001 b
Hemiptera	Reduviidae	3.3	0.056 ± 0.010 a	0.027 ± 0.003 b

**Table 4:** Key contributors and their contributions to the dissimilarity in community composition of ground-dwelling insects between large (*n* = 4) and small (*n* = 5) fragments embedded within tree plantations at Yachang Forest Farm, Guangxi, China. Different letters within the same row indicate significant differences in relative abundance between small and large fragments (*p* < 0.05). Data are presented as mean ± SE.

Order	Family	Contribution (%)	Relative Abundance	
			Large Fragment	Small Fragment
Blattodea	Blattellidae	34.6	0.407 ± 0.045 a	0.337 ± 0.034 b
Orthoptera	Gryllidae	21.0	0.204 ± 0.054 a	0.184 ± 0.033 b
Diptera	Drosophilidae	16.2	0.145 ± 0.035 a	0.121 ± 0.034 b
	Phoridae	2.0	0.016 ± 0.010 a	0.004 ± 0.002 b
Coleoptera	Mordellidae	1.4	0.012 ± 0.004 a	0.001 ± 0.004 b
	Staphylinidae	1.3	0.013 ± 0.004 a	0.012 ± 0.003 b

## Data Availability

The original contributions presented in this study are included in the article/Supplementary Material. Further inquiries can be directed to the corresponding author.

## Supplementary Materials

The following supporting information can be downloaded at: https://www.mdpi.com/article/10.3390/insects17040399/s1, Figure S1: Relative abundances of insect taxa at the order level in remnant natural forest fragments, intact natural forest (NF), and *Eucalyptus* (EP) and *Pinus massoniana* (PP) plantations at Yachang Forest Farm, Guangxi, China; Figure S2: Comparison of soil properties, litter properties and vegetation structure between low-isolation (*n* = 4) and high-isolation (*n* = 5) fragments embedded within tree plantations at Yachang Forest Farm, Guangxi, China; Figure S3: Non-metric multidimensional scaling (NMDS) ordination illustrating the effects of fragment isolation (low vs. high isolation) on tree (A) and seedling (B) community composition in nine remnant natural forest fragments embedded within tree plantations at Yachang Forest Farm, Guangxi, China; Figure S4: Comparison of soil properties, litter properties and vegetation structure between large (*n* = 4) and small (*n* = 5) fragments embedded within tree plantations at Yachang Forest Farm, Guangxi, China; Figure S5: Non-metric multidimensional scaling (NMDS) ordination illustrating the effects of forest fragment area (large vs. small) on tree (A) and seedling (B) community composition in nine remnant natural forest fragments embedded within tree plantations at Yachang Forest Farm, Guangxi, China; Figure S6: Comparison of γ-diversity between tree plantations (*Eucalyptus* and *Pinus massoniana* plantations) and remnant natural forest fragments, as well as between the intact natural forests and remnant fragments, without (A) and with (B) controlling for sampling effects at Yachang Forest Farm, Guangxi, China; Figure S7: Comparison of α-diversity between tree plantations (*Eucalyptus* and *Pinus massoniana* plantations) and remnant natural forest fragments, as well as between the intact natural forests and remnant fragments, without (A) and with (B) controlling for sampling effects at Yachang Forest Farm, Guangxi, China; Figure S8: Comparison of β-diversity between tree plantations (*Eucalyptus* and *Pinus massoniana* plantations) and remnant natural forest fragments, as well as between the intact natural forests and remnant fragments, without (A) and with (B) controlling for sampling effects at Yachang Forest Farm, Guangxi, China; Figure S9: Non-metric multidimensional scaling (NMDS) ordination illustrating the effects of forest types (remnant natural forest fragment, intact natural forest (NF), *Eucalyptus* (EP) plantation, and *Pinus massoniana* (PP) plantation) on the community composition of ground-dwelling insects at Yachang Forest Farm, Guangxi, China; Figure S10: Relative abundances of insect taxa at the family level in remnant natural forest fragments, intact natural forest (NF), and *Eucalyptus* (EP) and *Pinus massoniana* (PP) plantations at Yachang Forest Farm, Guangxi, China; Figure S11: Effects of fragment isolation (A, C, E) and area (B, D, F) on γ-, a-, β-diversity of ground-dwelling insects (after controlling for sampling effects) in nine remnant natural forest fragments surrounded by tree plantations at Yachang Forest Farm, Guangxi, China; Table S1: Characteristics of the nine remnant natural forest fragments, the intact natural forest (NF), the *Eucalyptus* (EP) and *Pinus massoniana* (PP) plantations at Yachang Forest Farm, Guangxi, China; Table S2: List of ground-dwelling insects captured by pitfall traps indicating the dominant species and their feeding habits for each family across different forest types (including nine remnant natural forest fragments, intact natural forest, *Eucalyptus* and *Pinus massoniana* plantations) at Yachang Forest Farm, Guangxi, China; Table S3: The total number of captures for each order for nine remnant natural forest fragments, the intact natural forest (NF), the *Eucalyptus* (EP) and *Pinus massoniana* (PP) plantations at Yachang Forest Farm, Guangxi, China; Table S4: Summary of stepwise regression models explaining variation in α-diversity in nine remnant natural forest fragments embedded within tree plantations at Yachang Forest Farm, Guangxi, China; Table S5: Summary of ordinary multiple linear regression models testing the effects of final selected explanatory variables (identified by stepwise regression in Table S4) on the α-diversity of ground-dwelling insects in nine remnant natural forest fragments embedded within tree plantations at Yachang Forest Farm, Guangxi, China.
