# Supplementary material for: Tree Plantation-Driven Forest Fragmentation Reduces Ground-Dwelling Insect Diversity Through Cascading Declines in Seedling Density
Source: Insects. 2026 Apr 7;17(4):399. doi: 10.3390/insects17040399 (PMC13115592; doi:10.3390/insects17040399)
Supplement: Supplementary file 1 [file insects-17-00399-s001.zip › insects-4200747-supplementary.pdf]

Supplementary Information for

Tree plantation-driven forest fragmentation reduces ground-dwelling insect diversity through cascading declines in seedling density

This word file includes:

Figure S1 to S11

Table S1 to S5

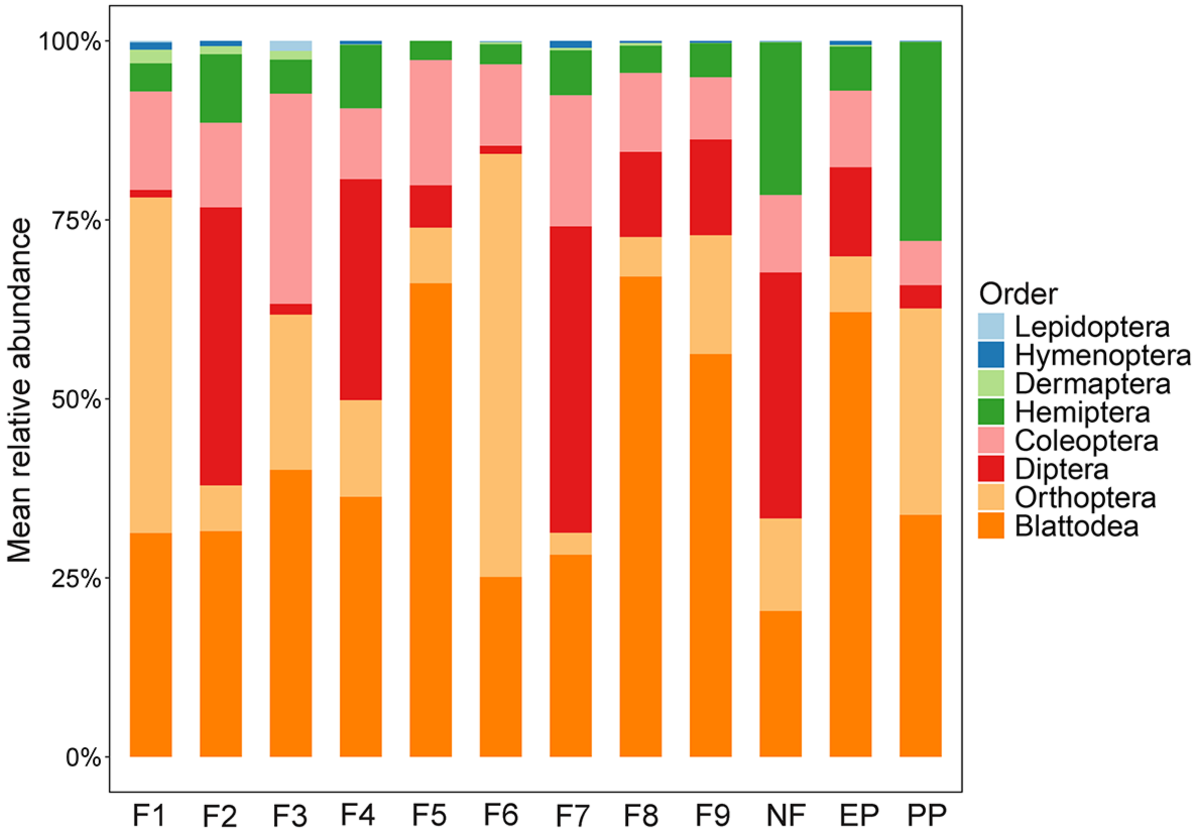

Figure S1 Relative abundances of insect taxa at the order level in remnant natural forest fragments, intact natural forest (NF), and *Eucalyptus* (EP) and *Pinus massoniana* (PP) plantations at Yachang Forest Farm, Guangxi, China. The remnant fragments (F1–F9) are ordered by size from smallest to largest.

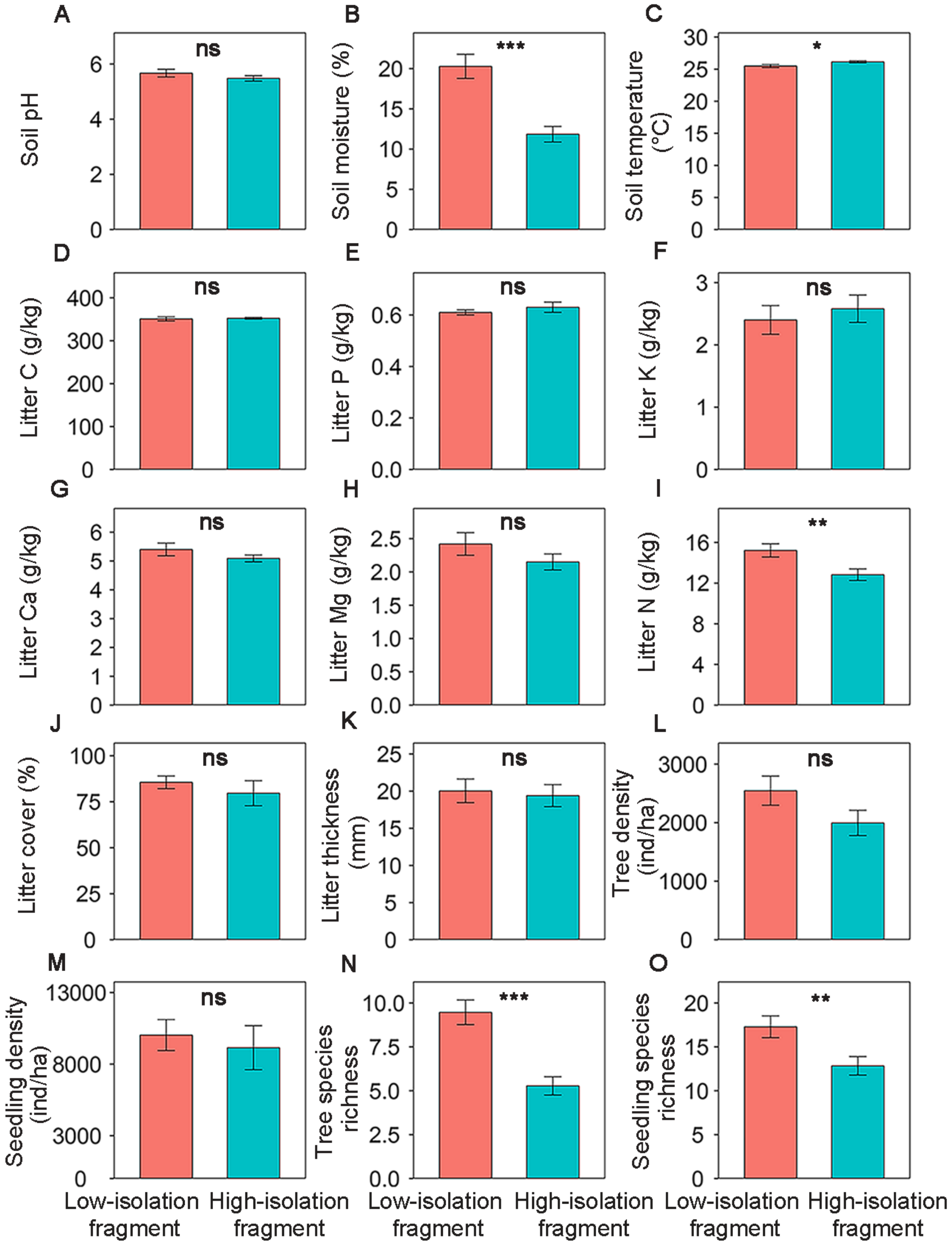

Figure S2 Comparison of soil properties, litter properties and vegetation structure between low-isolation (n = 4) and high-isolation (n = 5) fragments embedded within tree plantations at Yachang Forest Farm, Guangxi, China. ns indicates no significant difference between low-isolation and high-isolation fragments, *: $P < 0.05$, **: $0.001 \leq P < 0.01$, ***: $P < 0.001$.

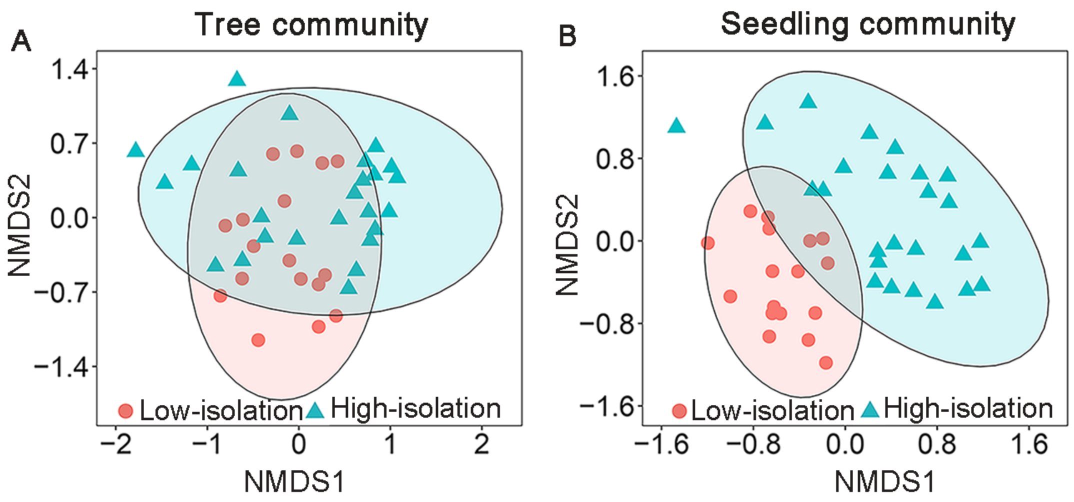

Figure S3 Non-metric multidimensional scaling (NMDS) ordination illustrating the effects of fragment isolation (low vs. high isolation) on tree (A) and seedling (B) community composition in nine remnant natural forest fragments embedded within tree plantations at Yachang Forest Farm, Guangxi, China. Red circles denote 95% confidence ellipses for low-isolation fragments, while blue circles denote 95% confidence ellipses for high-isolation fragments.

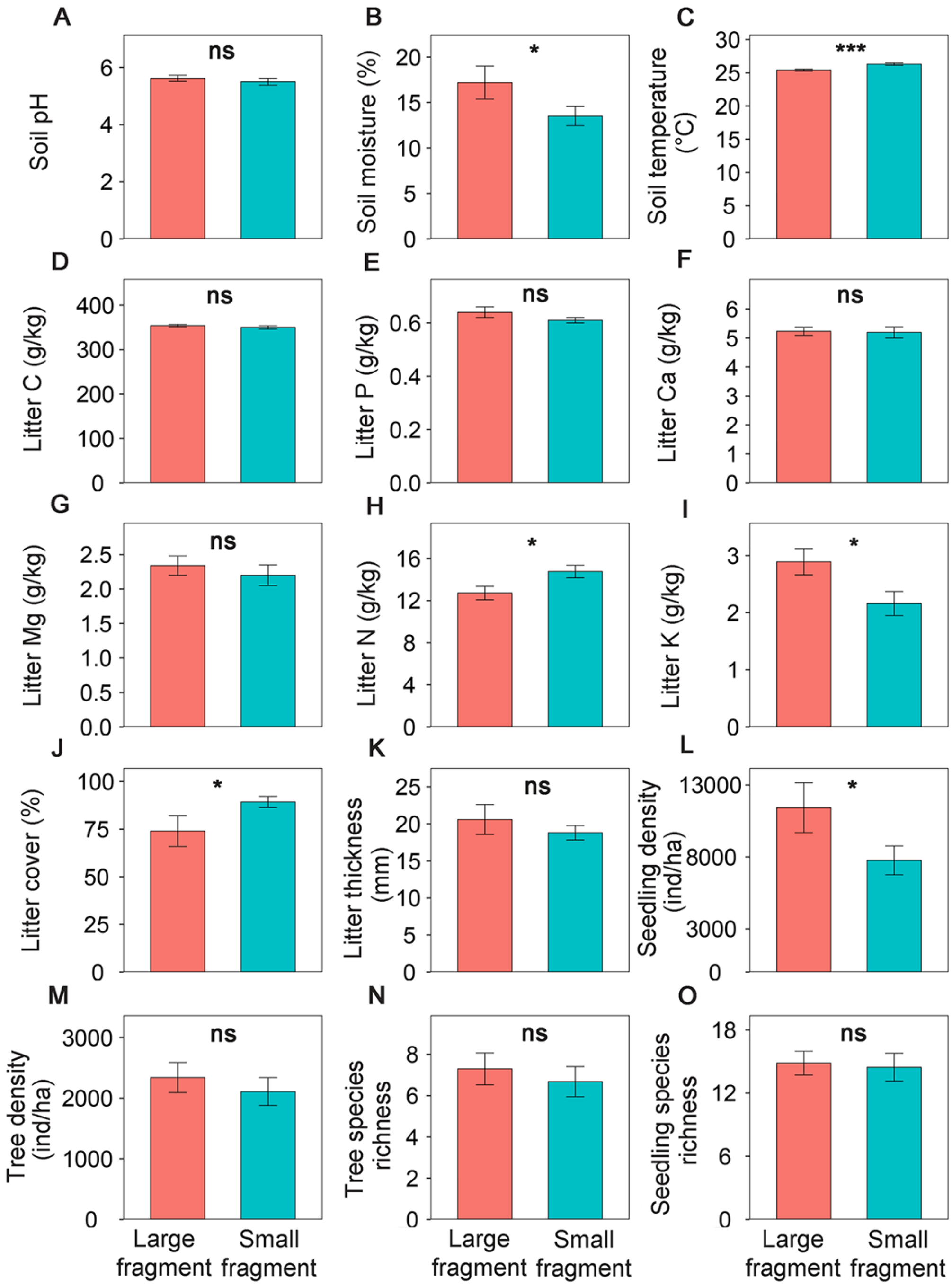

Figure S4 Comparison of soil properties, litter properties and vegetation structure between large (n = 4) and small (n = 5) fragments embedded within tree plantations at Yachang Forest Farm, Guangxi, China. ns indicates no significant difference between large and small fragments, *: $P < 0.05$, ***: $P < 0.001$.

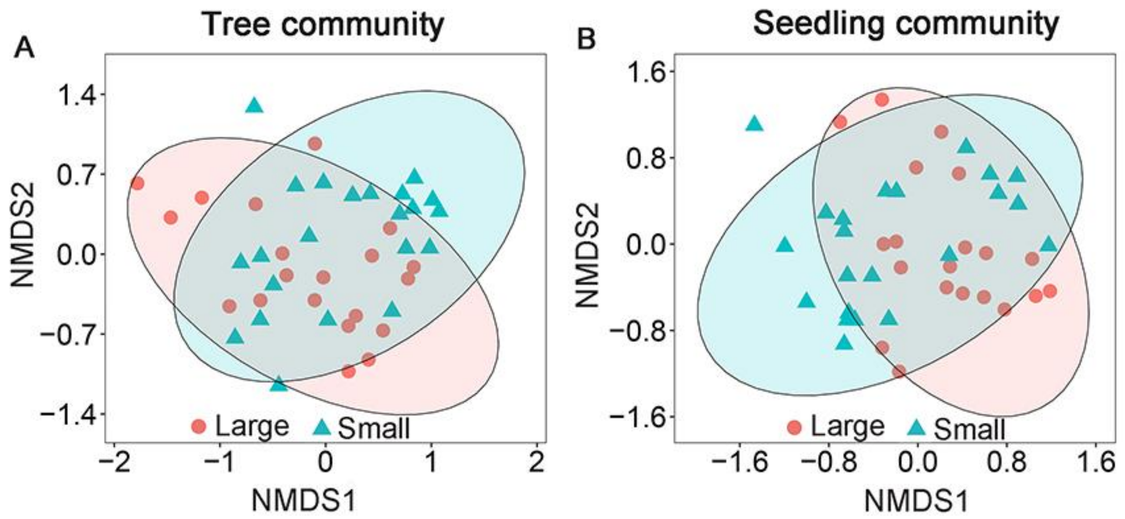

Figure S5 Non-metric multidimensional scaling (NMDS) ordination illustrating the effects of forest fragment area (large vs. small) on tree (A) and seedling (B) community composition in nine remnant natural forest fragments embedded within tree plantations at Yachang Forest Farm, Guangxi, China. Red circles denote 95% confidence ellipses for large fragments, while blue circles denote 95% confidence ellipses for small fragments.

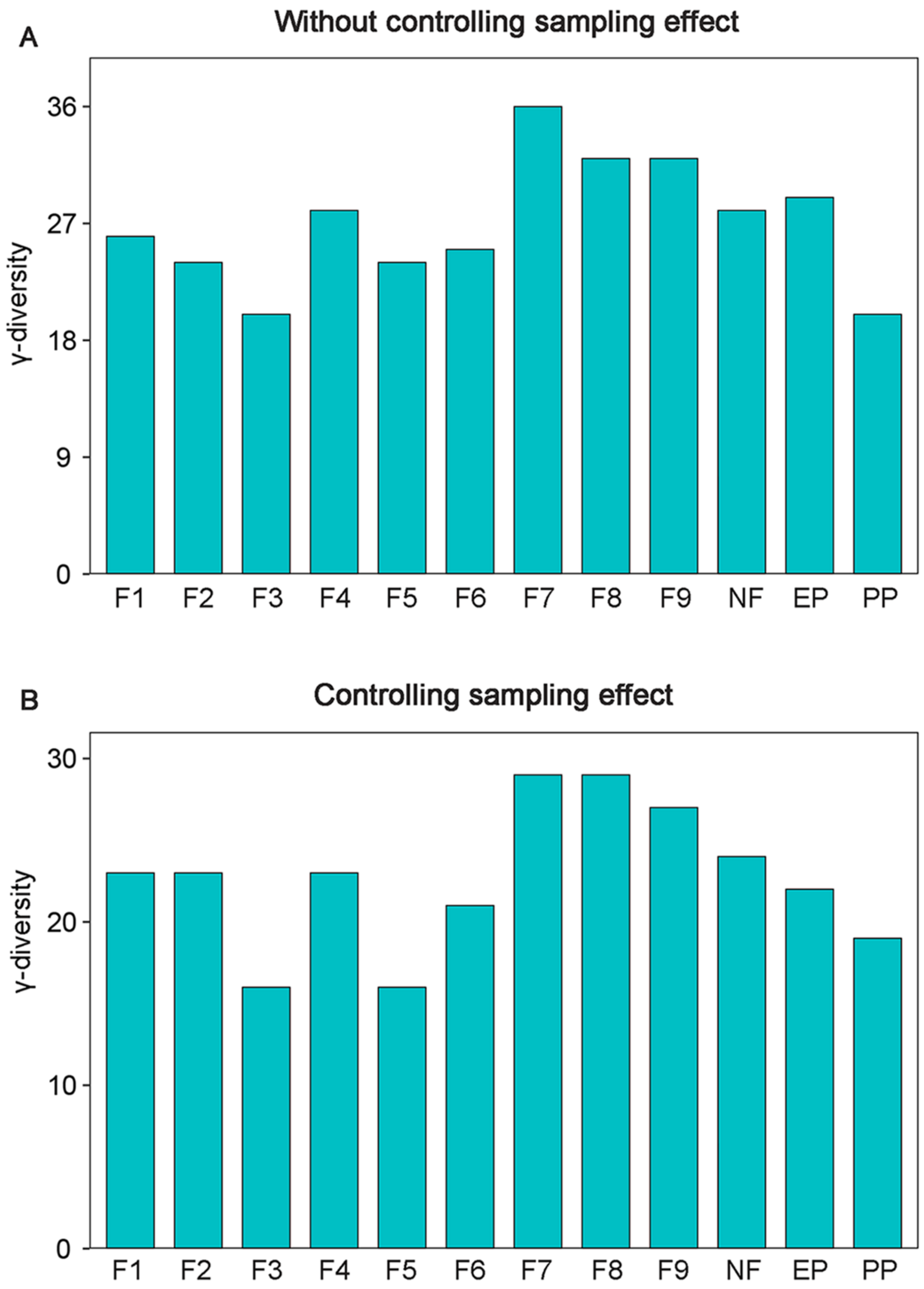

Figure S6 Comparison of γ-diversity between tree plantations (*Eucalyptus* and *Pinus massoniana* plantations) and remnant natural forest fragments, as well as between the intact natural forests and remnant fragments, without (A) and with (B) controlling for sampling effects at Yachang Forest Farm, Guangxi, China. The remnant fragments

(F1–F9) are ordered by size from smallest to largest. NF represents intact natural forest; EP represents *Eucalyptus* plantation; PP represents *Pinus massoniana* plantation.

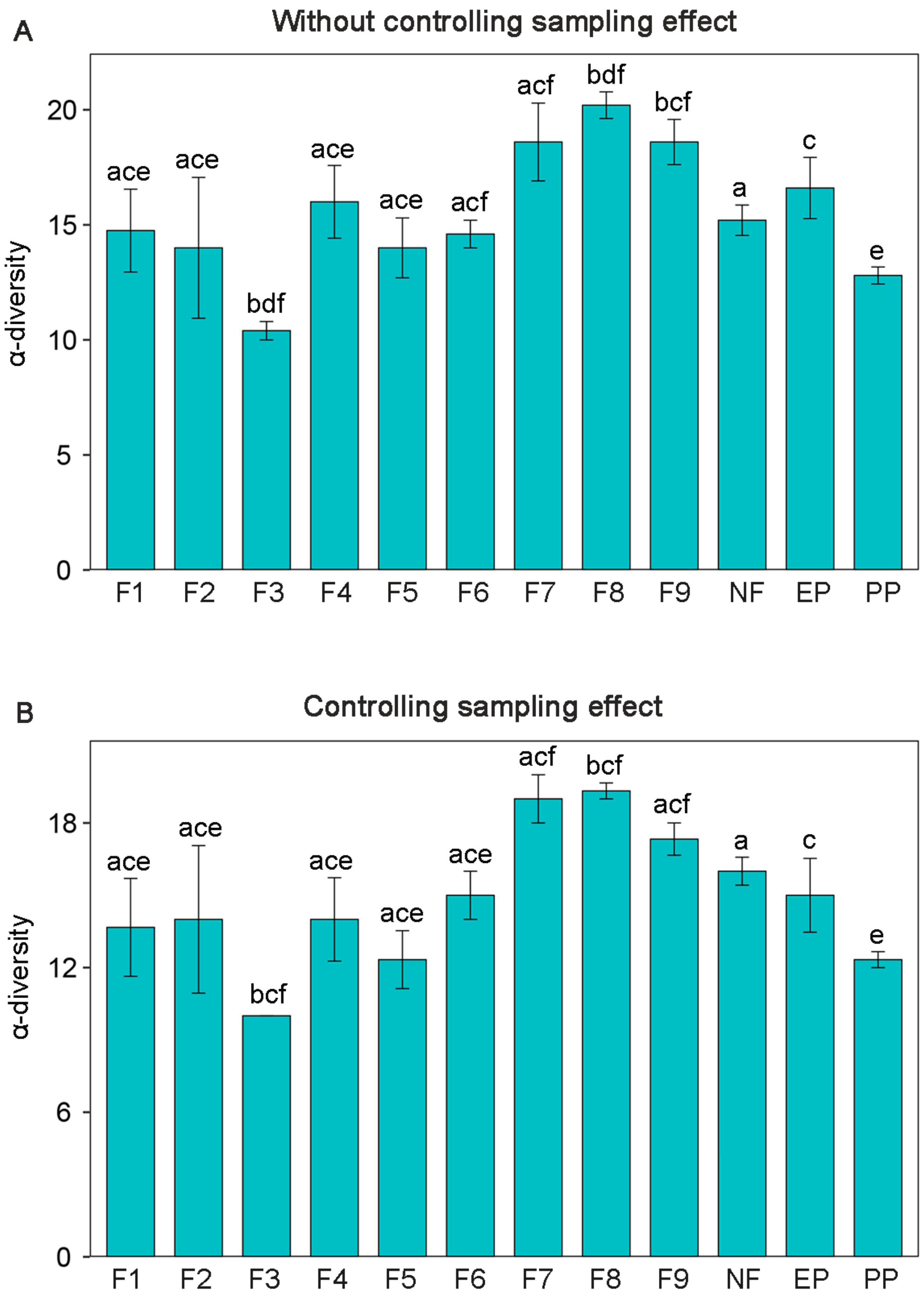

Figure S7 Comparison of α-diversity between tree plantations (*Eucalyptus* and *Pinus massoniana* plantations) and remnant natural forest fragments, as well as between the intact natural forests and remnant fragments, without (A) and with (B) controlling for sampling effects at Yachang Forest Farm, Guangxi, China. The remnant fragments (F1–F9) are ordered by size from smallest to largest. NF represents intact natural

forest; EP represents *Eucalyptus* plantation; PP represents *Pinus massoniana* plantation. Letter a and b indicate significant (P < 0.05) differences between the intact natural forests(NF) and remnant natural forest fragments; letter c and d indicate significant (P < 0.05) differences between *Eucalyptus* plantations (EP) and remnant natural forest fragments; letter e and f within each column indicate significant (P < 0.05) differences between *Pinus massoniana* plantations(PP) and remnant natural forest fragments.

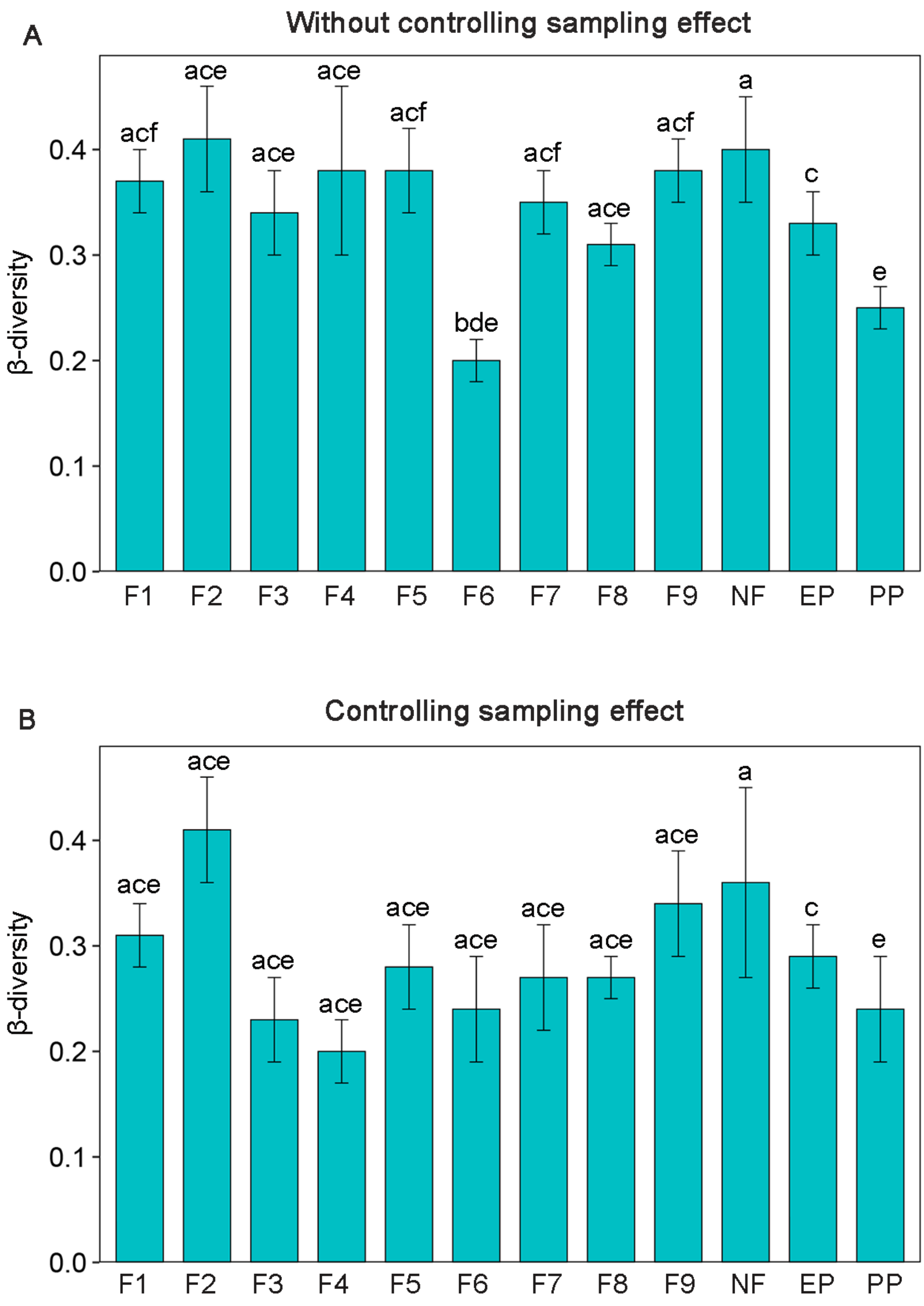

Figure S8 Comparison of β-diversity between tree plantations (*Eucalyptus* and *Pinus massoniana* plantations) and remnant natural forest fragments, as well as between the intact natural forests and remnant fragments, without (A) and with (B) controlling for sampling effects at Yachang Forest Farm, Guangxi, China. The remnant fragments (F1–F9) are ordered by size from smallest to largest. NF represents intact natural

forest; EP represents *Eucalyptus* plantation; PP represents *Pinus massoniana* plantation. Letter a and b indicate significant (P < 0.05) differences between the intact natural forests(NF) and remnant natural forest fragments; letter c and d indicate significant (P < 0.05) differences between *Eucalyptus* plantations (EP) and remnant natural forest fragments; letter e and f within each column indicate significant (P < 0.05) differences between *Pinus massoniana* plantations(PP) and remnant natural forest fragments.

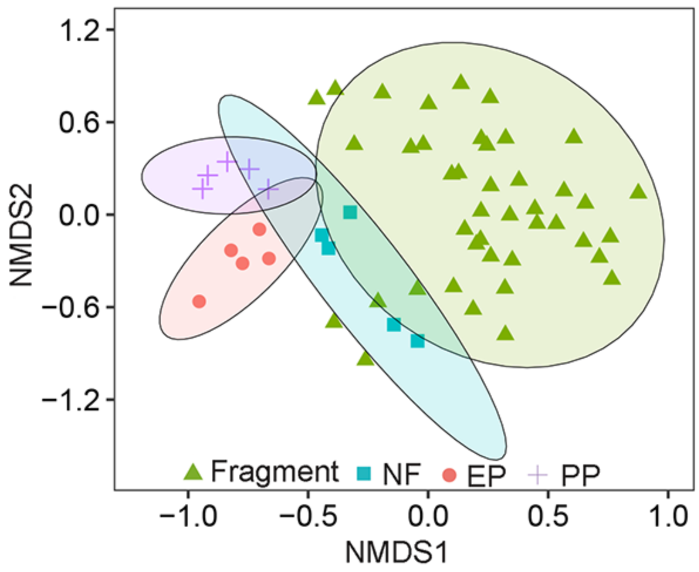

Figure S9 Non-metric multidimensional scaling (NMDS) ordination illustrating the effects of forest types (remnant natural forest fragment, intact natural forest (NF), *Eucalyptus* (EP) plantation, and *Pinus massoniana* (PP) plantation) on the community composition of ground-dwelling insects at Yachang Forest Farm, Guangxi, China. Green circles denote 95% confidence ellipses for fragments, blue circles denote 95% confidence ellipses for intact natural forest, red circles denote 95% confidence ellipses for *Eucalyptus* plantation, and purple circles denote 95% confidence ellipses for *Pinus massoniana* plantation.

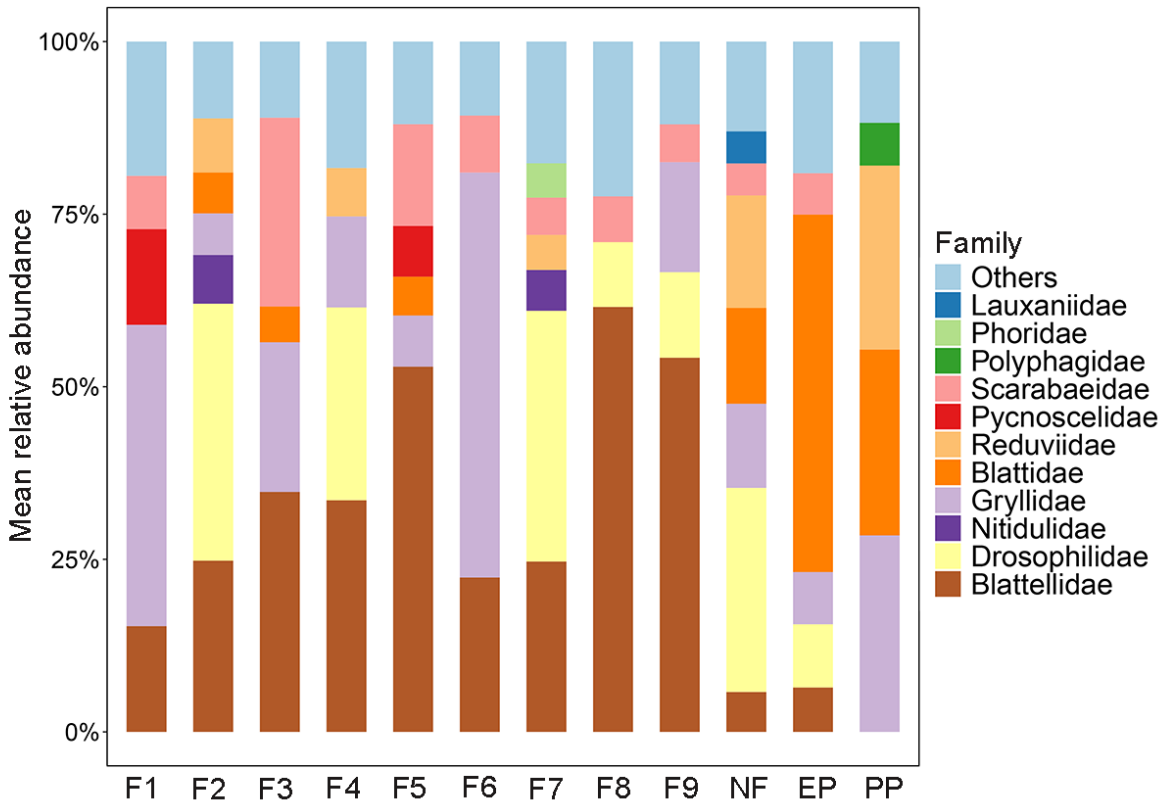

Figure S10 Relative abundances of insect taxa at the family level in remnant natural forest fragments, intact natural forest (NF), and *Eucalyptus* (EP) and *Pinus massoniana* (PP) plantations at Yachang Forest Farm, Guangxi, China. The remnant fragments (F1–F9) are ordered by size from smallest to largest. Only families with a mean relative abundance ≥ 5% across all samples are shown, while all other taxa are grouped as "others".

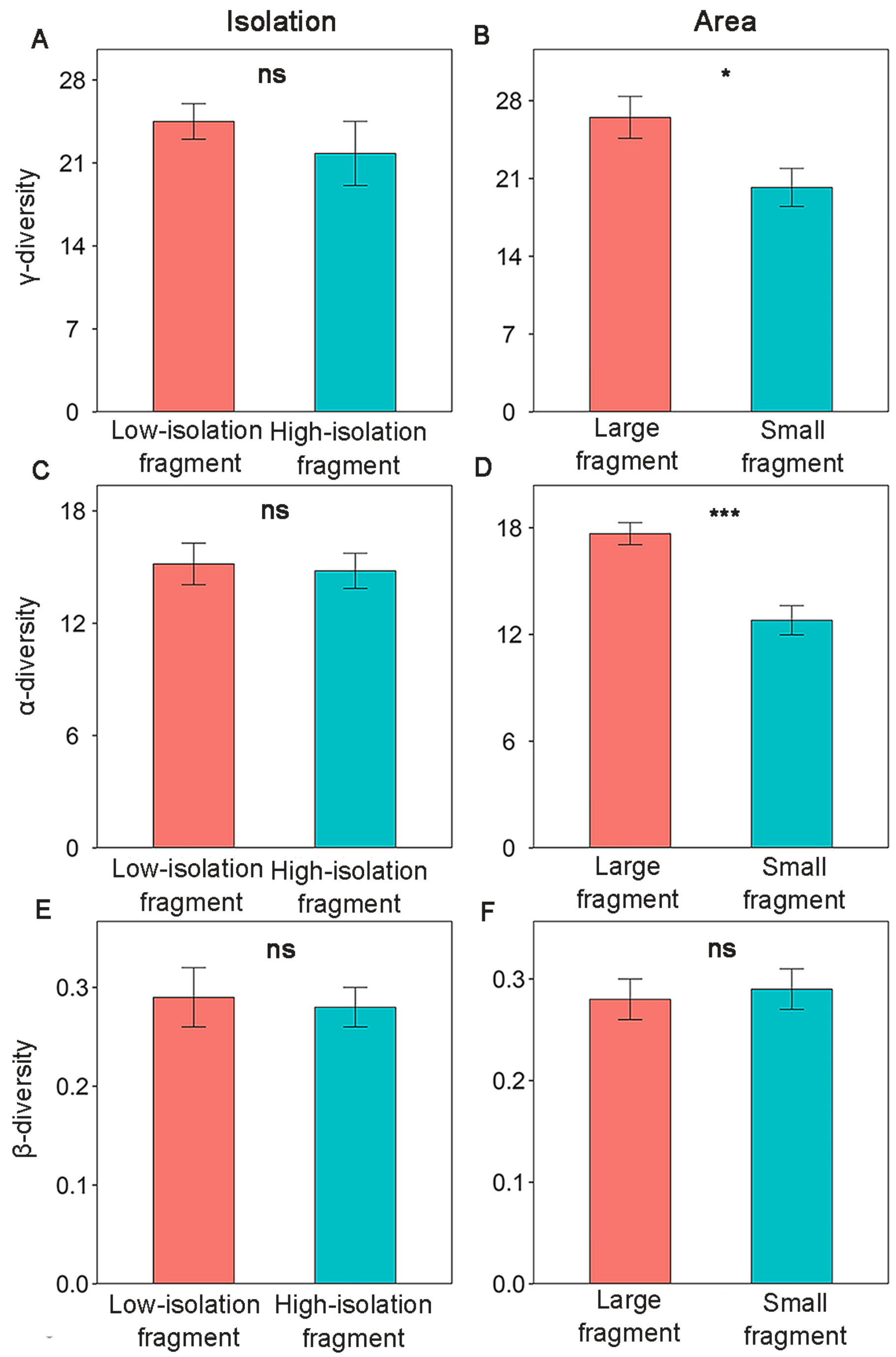

Figure S11 Effects of fragment isolation (A, C, E) and area (B, D, F) on γ-, a-, β-diversity of ground-dwelling insects (after controlling for sampling effects) in nine remnant natural forest fragments surrounded by tree plantations at Yachang Forest Farm, Guangxi, China. ns indicates no significant difference between fragment categories, *: $P < 0.05$, ***: $P < 0.001$. $n = 4$ and 5 for low-isolation and high-

isolation fragments, respectively. *n* = 4 and 5 for large and small fragments, respectively.

Table S1 Characteristics of the nine remnant natural forest fragments, the intact natural forest (NF), the *Eucalyptus* (EP) and *Pinus massoniana* (PP) plantations at Yachang Forest Farm, Guangxi, China. F1–F9 (ranked according to their size from the smallest to the largest) represent the studied remnant natural forest fragments. DNR: the distance to the nearby nature reserve. Low & High: low-isolation and high-isolation fragments. Small & Large: small and large fragments.

Forest type	Area (m^2)	Area type	Latitude (N)	Longitude (E)	Elevation (m)	Canopy cover (%)	Slope (°)	DNR (m)	DNR type
F1	9782.2	Small	24°52'3.32"	106°14'28.96"	685	80	33	4125.6	Low
F2	13636.5	Small	24°49'36.88"	106°16'36.84"	607	90	31	1753.1	Low
F3	16634.6	Small	24°54'49.97"	106°12'31.32"	697	80	29	12791.1	High
F4	27688.8	Small	24°49'51.66"	106°16'15.64"	522	80	25	2484.0	Low
F5	32706.8	Small	24°52'55.92"	106°13'49.22"	532	80	18	8941.2	High
F6	45484.1	Large	24°54'52.02"	106°12'17.17"	696	75	26	13194.2	High
F7	45530.1	Large	24°49'12.23"	106°17'42.07"	772	85	28	22.5	Low
F8	47109.9	Large	24°52'35.35"	106°13'37.22"	640	75	24	8449.6	High
F9	76899.5	Large	24°52'45.84"	106°13'48.11"	586	80	23	8610.4	High
NF			24°48'14.41"–24°53'56.43"	106°11'31.24"–106°27'3.63"	779–991	85–90	21–35		
EP			24°50'56.44"–24°56'42.10"	106°12'18.05"–106°16'30.11"	668–857	75–85	14–27		
PP			24°49'22.83"–24°55'33.66"	106°11'16.86"–106°16'22.87"	542–913	80–85	17–30		

Table S2 List of ground-dwelling insects captured by pitfall traps indicating the dominant species and their feeding habits for each family across different forest types (including nine remnant natural forest fragments, intact natural forest, *Eucalyptus* and *Pinus massoniana* plantations) at Yachang Forest Farm, Guangxi, China.

Order	Family	Dominant species	Feeding habits
Blattodea	Blattellidae	*Blattella germanica*	Detritivorous
	Blattidae	*Blatta orientalis*	Detritivorous
	Pycnoscelidae	*Pycnoscelus surinamensis*	Detritivorous
	Polyphagidae	*Eupolyphaga sinensis*	Detritivorous
	Epilampridae	*Rhabdoblatta karnyi*	Detritivorous
	Blaberidae		Detritivorous
Orthoptera	Gryllidae	*Loxoblemmus arietulus*	Detritivorous
	Catantopidae	*Xenocatantops humilis*	Phytophagous
	Acrididae	*Phlaeoba infumata*	Phytophagous
	Gryllotalpidae	*Gryllotalpa orientalis*	Detritivorous
	Eumastacidae	*China mantispoides*	Phytophagous
	Rhaphidophoridae		Phytophagous
	Myrmecophilidae		Predator
	Tetrigidae		Phytophagous
Diptera	Drosophilidae	*Drosophila immigrans*	Omnivorous
	Phoridae	*Megaselia scalaris*	Omnivorous
	Lauxaniidae	*Homoneura euaresta*	Omnivorous
	Agromyzidae	*Phytomyza ranunculi*	Phytophagous
	Calliphoridae	*Lucilia caesar*	Omnivorous
	Trypetidae	*Rhagoletis alternata*	Predator (Parasitoids)
	Dolichopodidae		Predator
	Tachinidae		Phytophagous

	Simuliidae		Phytophagous
	Sciaridae		Detritivorous
	Milichiidae		Detritivorous
	Anthomyiidae		Omnivorous
	Empididae		Phytophagous
	Chironomidae		Omnivorous
	Muscidae		Omnivorous
Coleoptera	Scarabaeidae	*Onthophagus vacca*	Detritivorous
	Nitidulidae	*Omosita depressa*	Omnivorous
	Staphylinidae	*Paederus fuscipes*	Predator
	Mordellidae	*Scirtes* sp.	Omnivorous
	Curculionidae	*Sipalinus gigas*	Phytophagous
	Chrysomelidae	*Aulacophora indica*	Phytophagous
	Carabidae	*Pterostichus adstrictus*	Predator
	Elateridae	*Ampedus sanguinolentus*	Phytophagous
	Erotylidae	*Amblyopus vittatus*	Detritivorous
	Cleridae	*Necrobia ruficollis*	Predator
	Laemophloeidae		Phytophagous
	Coccinellidae		Predator
	Scolytidae	*Xylosandrus crassiusculus*	Predator (Parasitoids)
	Cerambycidae		Phytophagous
	Histeridae		Predator
	Lucanidae		Phytophagous
Hemiptera	Reduviidae	*Rhynocoris annulatus*	Predator
	Coreidae	*Homoeocerus* sp.	Phytophagous
	Cydnidae	*Adrisa magna*	Phytophagous
	Cicadellidae	*Oides epipleuralis*	Phytophagous
	Lygaeidae	*Metochus abbreviatus*	Phytophagous
	Pentatomidae	*Rhacognathus punctatus*	Phytophagous

	Cicadidae		Phytophagous
	Issidae		Phytophagous
	Cercopidae		Phytophagous
Hymenoptera	Diapriidae	*Trichopria* sp.	Predator (Parasitoids)
	Pteromalidae	*Callitula bicolor*	Predator (Parasitoids)
	Eumenidae		Phytophagous
	Ichneumonidae	*Xanthopimpla* sp.	Predator (Parasitoids)
	Sphecidae		Predator
	Apidae		Phytophagous
	Elasmidae		Predator (Parasitoids)
	Eulophidae		Predator (Parasitoids)
	Braconidae		Predator (Parasitoids)
	Bombidae		Omnivorous
Dermaptera	Anisolabididae	*Euborellia annulipes*	Predator
	Labiduridae	*Eparchus insignis*	Predator
Lepidoptera	Crambidae	*Glyphodes stolalis*	Phytophagous

Table S3 The total number of captures for each order for nine remnant natural forest fragments, the intact natural forest (NF), the *Eucalyptus* (EP) and *Pinus massoniana* (PP) plantations at Yachang Forest Farm, Guangxi, China. F1–F9 (ranked according to their size from the smallest to the largest) represent the studied remnant natural forest fragments.

Forest	Blattodea	Orthoptera	Diptera	Coleoptera	Hemiptera	Hymenoptera	Dermaptera	Lepidoptera
F1	163	234	5	69	18	5	9	1
F2	97	25	127	47	42	3	4	0
F3	277	141	11	174	31	0	7	9
F4	472	186	415	109	127	8	1	0
F5	751	101	66	189	29	0	0	0
F6	432	1000	19	195	48	1	5	2
F7	376	48	635	260	96	15	5	0
F8	1333	103	246	195	68	6	6	0
F9	1506	394	410	261	127	8	1	0
NF	333	212	428	144	337	1	1	1
EP	490	62	94	82	51	4	1	0
PP	519	420	51	93	414	2	1	0
Total	6749	2926	2507	1818	1388	53	41	13

Table S4 Summary of stepwise regression models explaining variation in α-diversity in nine remnant natural forest fragments embedded within tree plantations at Yachang Forest Farm, Guangxi, China. The plant metric was represented by species richness.

Variable	R^2	ΔR^2	ΔAIC
Full model	0.348	0	0
-Soil pH	0.337	0.011	-1.28
-Soil moisture	0.348	0.000	-1.97
-Soil temperature	0.345	0.003	-1.79
-Litter C	0.331	0.017	-0.87
-Litter N	0.323	0.025	-0.39
-Litter P	0.32	0.028	-0.20
-Litter K	0.343	0.005	-1.68
-Litter Ca	0.321	0.027	-0.30
-Litter Mg	0.336	0.012	-1.21
-Litter cover	0.322	0.026	-0.32
-Litter thickness	0.346	0.002	-1.87
-Tree richness	0.278	0.070	2.31
-Seedling richness	0.348	0.000	-1.95

Note: The full model included all explanatory variables. Symbols are defined as follows: “-” in the first column indicates a variable excluded by stepwise selection; ΔAIC (Akaike information criterion) and ΔR^2 represent the differences in AIC and model R^2, respectively, relative to the full model.

Table S5 Summary of ordinary multiple linear regression models testing the effects of final selected explanatory variables (identified by stepwise regression in Table S4) on the α-diversity of ground-dwelling insects in nine remnant natural forest fragments embedded within tree plantations at Yachang Forest Farm, Guangxi, China. The plant metric was represented by species richness.

Variable	Estimate	SE	*t*	*P*	Partial R^2
**α-diversity: R^2 = 0.129, *P* = 0.020**					
Tree richness	0.36	0.15	2.43	0.020	0.129
